# Human post-mortem organotypic brain slice cultures: a tool to study pathomechanisms and test therapies

**DOI:** 10.1186/s40478-024-01784-1

**Published:** 2024-05-31

**Authors:** Bonnie C. Plug, Ilma M. Revers, Marjolein Breur, Gema Muñoz González, Jaap A. Timmerman, Niels R.C. Meijns, Daniek Hamberg, Jikke Wagendorp, Erik Nutma, Nicole I. Wolf, Antonio Luchicchi, Huibert D. Mansvelder, Niek P. van Til, Marjo S. van der Knaap, Marianna Bugiani

**Affiliations:** 1 Department of Paediatrics and Child Neurology, Emma Children’s Hospital, Amsterdam University Medical Centre, Meibergdreef 9, Amsterdam, 1100 DD The Netherlands; 2 Amsterdam Leukodystrophy Center, Emma Children’s Hospital, Amsterdam University Medical Centre, Amsterdam Neuroscience, Cellular & Molecular Mechanisms, Meibergdreef 9, 1100 DD Amsterdam, The Netherlands; 3 Department of Anatomy and Neurosciences, MS Center Amsterdam, Amsterdam University Medical Centre, VU University, Amsterdam Neuroscience, De Boelelaan 1108, Amsterdam, 1081 HZ The Netherlands; 4 Department of Integrative Neurophysiology, Center for Neurogenomics and Cognitive Research, VU University, Amsterdam Neuroscience, De Boelelaan 1085, Amsterdam, 1081 HV The Netherlands; 5 Department of Pathology, Amsterdam Neuroscience, Amsterdam University Medical Centre, Meibergdreef 9, Amsterdam, 1100 DD The Netherlands

**Keywords:** Human post-mortem brain, Organotypic brain slice culture, Leukodystrophy, White matter, Gene therapy

## Abstract

Human brain experimental models recapitulating age- and disease-related characteristics are lacking. There is urgent need for human-specific tools that model the complex molecular and cellular interplay between different cell types to assess underlying disease mechanisms and test therapies. Here we present an adapted ex vivo organotypic slice culture method using human post-mortem brain tissue cultured at an air-liquid interface to also study brain white matter. We assessed whether these human post-mortem brain slices recapitulate the in vivo neuropathology and if they are suitable for pathophysiological, experimental and pre-clinical treatment development purposes, specifically regarding leukodystrophies. Human post-mortem brain tissue and cerebrospinal fluid were obtained from control, psychiatric and leukodystrophy donors. Slices were cultured up to six weeks, in culture medium with or without human cerebrospinal fluid. Human post-mortem organotypic brain slice cultures remained viable for at least six weeks ex vivo and maintained tissue structure and diversity of (neural) cell types. Supplementation with cerebrospinal fluid could improve slice recovery. Patient-derived organotypic slice cultures recapitulated and maintained known in vivo neuropathology. The cultures also showed physiologic multicellular responses to lysolecithin-induced demyelination ex vivo, indicating their suitability to study intrinsic repair mechanisms upon injury. The slice cultures were applicable for various experimental studies, as multi-electrode neuronal recordings. Finally, the cultures showed successful cell-type dependent transduction with gene therapy vectors. These human post-mortem organotypic brain slice cultures represent an adapted ex vivo model suitable for multifaceted studies of brain disease mechanisms, boosting translation from human ex vivo to in vivo. This model also allows for assessing potential treatment options, including gene therapy applications. Human post-mortem brain slice cultures are thus a valuable tool in preclinical research to study the pathomechanisms of a wide variety of brain diseases in living human tissue.

**Supplementary Information:**

The online version contains supplementary material available at 10.1186/s40478-024-01784-1.

## Introduction

Neurological disorders show involvement of different neural cell types that, in cell-autonomous and non-cell-autonomous ways, impact on brain function. One example are the leukodystrophies. Leukodystrophies are inherited disorders characterized by predominant involvement of the central nervous system (CNS) white matter. They arise due to defects in any of the white matter structural components, including astrocytes, microglia, axons, blood vessels, oligodendrocytes and/or myelin [19, 46]. To assess the underlying human-specific, complex disease mechanisms and test therapeutic strategies, there is urgent need for tools that recapitulate the intricate spatial, molecular and cellular interplay between all CNS cell types.

Transgenic mouse models have been of great value in unraveling brain disease mechanisms, also of leukodystrophies [8, 15, 17]. However, animal models replicating full clinical and pathological patients’ phenotype remain unachievable due to phylogenetic differences between mice and humans. In vitro models, including cultures of primary cells or induced pluripotent stem cells (iPSCs), reduce the use of laboratory animals and advance our knowledge on some cellular defects involved [15, 27]. However, they fail to represent the complex three-dimensional (3D) organization and interactions of various cell types in the mature brain. iPSC-based organoids come closer to recapitulate complex tissue, but for instance still lack blood vessels. Moreover, iPSCs lack part of the important biological information contributing to the disease cellular phenotype, including epigenetic and aging signatures [13, 16]. Organotypic brain slice cultures (OSCs) could be very useful in overcoming these limitations. OSCs are relatively thick slices of brain tissue rapidly dissected that can be cultured for several weeks ex vivo [21]. Here, all neural cell types are present and their histoarchitectural 3D organization remains preserved, closely resembling in vivo conditions [21]. Stoppini et al. [43]. established a method for culturing rodent brain slices at the air-liquid interface using semi-permeable membranes. This method has been widely applied to rodents of all ages [3, 6, 23, 24, 38, 52].

Several protocols have been established to culture slices of human brain tissue, mostly from surgically resected material [2, 18, 37, 39, 45]. While having the advantage of using tissue from live donors, this method is limited to specific patients, especially those with brain tumours and pharmacotherapy-resistant epilepsy, and is consequently also limited to specific brain regions. Additionally, these models are often optimized for neuronal electrophysiology studies [2, 18, 39, 45]. Only a few studies by Swaab et al. report successful culturing of human post-mortem neocortical brain (HPMB) slices in a free-floating fashion [30, 34, 35, 47, 48]. These slices were viable for several weeks ex vivo. Their use has, however, not been widely adopted and, due to the limited inclusion of white matter, optimized methods to study live cells in normal and diseased white matter are still lacking. Additionally, culturing HPMB tissue at the air-liquid interface has not yet been attempted.

In this proof-of-principle study, we developed an adapted ex vivo OSC method for culturing HPMB tissue at the air-liquid interface using tissue from control, psychiatric and leukodystrophy donors with the aim to also allow studies of the white matter. We evaluated HPMB-OSCs for long-term viability and assessed the effect of adding human cerebrospinal fluid (hCSF) [39]. We also assessed whether HPMB-OSCs recapitulate the in vivo neuropathology and assessed if they are suitable for pathophysiological, experimental and pre-clinical treatment development purposes. To this end, we evaluated whether HPMB-OSCs were amenable to test therapies, including gene therapy, and their response to injury. We conclude that our adapted HPMB–OSC method is applicable to brain white matter regions, with the potential to be widely adopted to study any brain disorder from any brain region from donors of any age.

## Materials and methods

### Human post-mortem brain tissue

HPMB tissue was obtained at autopsy from six leukodystrophy patients and one multiple sclerosis (MS) patient, classified as neuropathological disease donors; four psychiatric disorder donors; and four control donors who died of an unrelated medical condition without confounding morphologic neuropathology. Donor characteristics are presented in Table 1. The tissue collected from the MS patient and psychiatric donors was macroscopically and microscopically devoid of any pathological morphologic abnormalities. The diagnoses of the leukodystrophy patients were DNA-confirmed and included vanishing white matter (VWM), Pelizaeus-Merzbacher disease (PMD), metachromatic leukodystrophy (MLD), adult-onset leukoencephalopathy with axonal spheroids and pigmented glia (ALSP), hypomyelination due to a *TUBB2A* defect (HYPO), and Alexander disease (AxD). These are different leukodystrophies [46]. The study was approved by the local Medical Ethical Committee of the VU University Medical Center (Amsterdam, The Netherlands) and all experiments were performed in accordance with the declaration of Helsinki. For all donors, a written informed (parental) consent for a brain autopsy, the use of the medical data and permission to use the tissue for experimental purposes was obtained by the Amsterdam Leukodystrophy Center Biobank (https://www.amsterdamumc.org/en/research/knowledge-centers/leukodystrophy-center.htm) or the Netherlands Brain Bank (https://www.brainbank.nl/). Tissue collection was performed on average within 4.5 h post-mortem at the Amsterdam University Medical Center. A tissue block from the middle frontal gyrus containing about 40% cortex and 60% subcortical white matter was rapidly dissected and immersed in ice-cold dissection medium, consisting of Hibernate-A (Thermo Fisher, A1247501) and 1% penicillin-streptomycin (Thermo Fisher, 15140122). If obtainable, hCSF was collected post-mortem from the lateral ventricles using a 50 ml syringe and BD® 20Gx3.5” spinal needle.

**Table 1:** Donors’ demographic, clinical and genetic characteristics

Donor ID	Sex	Diagnosis	Gene variants	Age at death	Cause of death	P-M-D (hours)	pH of hCSF^a^
**Neuropathological disease donors**							
VWM (VWM492)	F	Vanishing white matter	*EIF2B5*, c.271 A > G, p.(Thr91Ala) and c.1745 + 5G > A, p.(Tyr583X)	61 y	Disease progression	4.0	n.a.^b^
PMD (H342)	M	Pelizaeus-Merzbacher disease	*PLP1* duplication	14 y	Disease progression	4.0	8.6
MLD	F	Metachromatic leukodystrophy	*ARSA*, c.1277 C > T, p.(Pro426Leu) and c.1130 C > T, p.(Pro377Leu)	11 y	Late GvHD following HSCT at 7 y	3.0	8.2
ALSP	M	Adult-onset leukoencephalopathy with axonal spheroids and pigmented glia	*CSF1R*, c.2329 C > T, p.(Arg777Trp)	50 y	Disease progression	4.0	8.2
HYPO	F	Hypomyelination	*TUBB2A*, c.1090_1092 del, p.(Ser364del), de novo	3 y	Disease progression and pneumonia	4.0	7.6
AxD	F	Alexander disease	*GFAP*, c.223G > A, p.(Glu75Lys), de novo	69 y	Disease progression	3.5	7.6
MS	F	Multiple Sclerosis	-	69 y	Rectal cancer	6.0	6.5
**Psychiatric disorder donors**							
PSY1	F	Post-traumatic stress disorder	-	60 y	Legal euthanasia	4.0	6.8
PSY2	F	Bipolar disorder	-	41 y	Legal euthanasia	5.5	6.4
PSY3	M	Bipolar disorder	-	63 y	Legal euthanasia	5.5	6.6
PSY4	F	Chronic fatigue syndrome, COPD	-	64 y	Lung emphysema	6.0	6.4
**Control donors**							
CTRL1	M	Non-demented control, COPD	-	94 y	Respiratory insufficiency due to COPD	4.5	6.6
CTRL2	F	Femur fracture	-	88 y	Legal euthanasia	4.0	6.7
CTRL3	F	Non-demented control, Endocrine disease	-	92 y	Heart failure	4.5	6.3
CTRL4	M	Non-demented control	-	58 y	Out-of-hospital cardiac arrest	4.0	n.a.^b^

*ARSA* = arylsulfatase A, *COPD* = chronic obstructive pulmonary disease, *CSF1R* = colony stimulating factor 1 receptor, *CTRL* = control, *EIF2B* = eukaryotic translation initiation factor 2B, *hCSF* = human cerebrospinal fluid, *F* = female, *GFAP* = glial fibrillary acidic protein, *GvHD* = graft-versus-host disease, *HSCT* = hematopoietic stem cell transplantation, *M* = male, n.a. = not available, *PLP1* = proteolipid protein 1, *P-M-D* = post-mortem delay, *PSY* = psychiatric disorder, *TUBB2A* = tubulin beta 2B class IIa, *y* = years

^a^ CSF pH measurements were performed after one freeze-thaw cycle for the leukodystrophy patients and immediately after collection for all other donors

^b^ No CSF pH measurement available

### Human post-mortem organotypic slice cultures

**Fig. 1:**
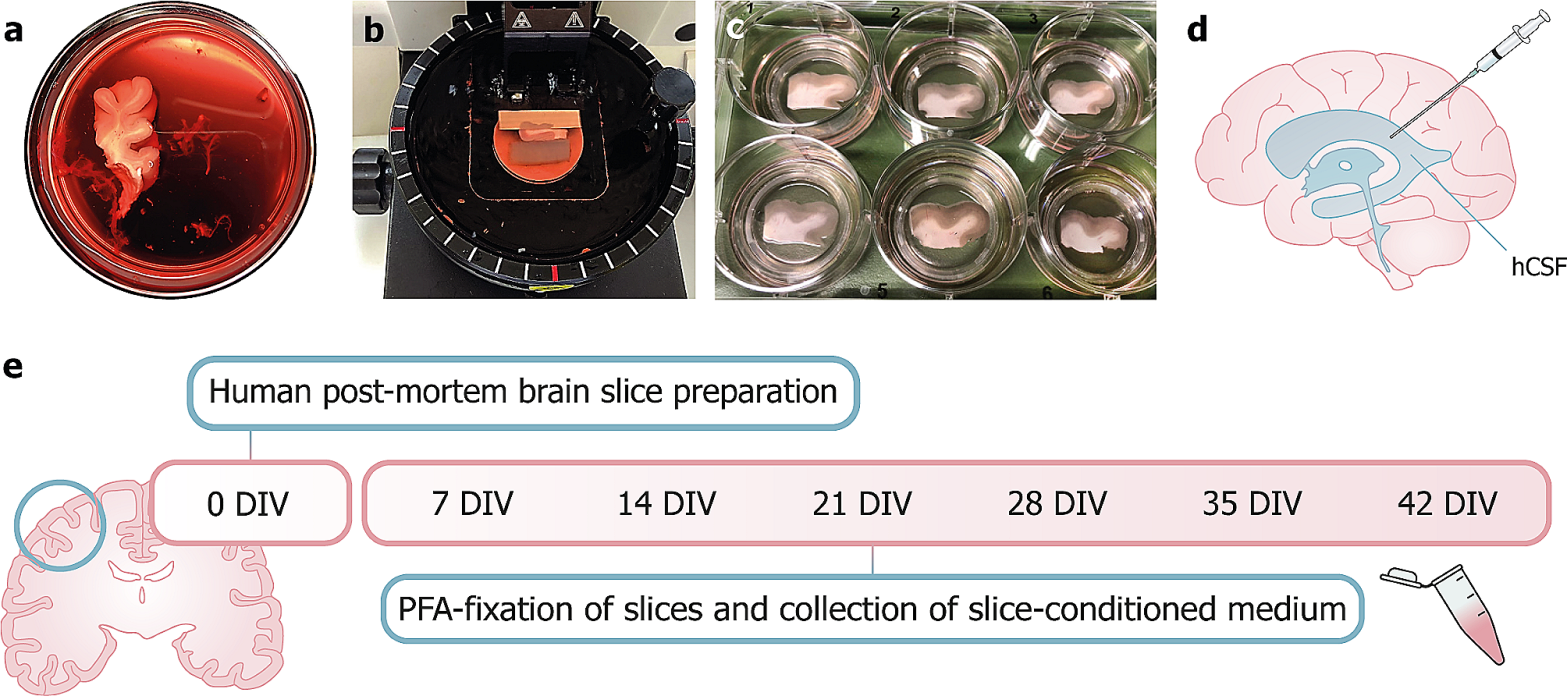
Preparation of HPMB-OSCs. (**a**) A tissue block of the middle frontal gyrus is dissected during autopsy and cut into a block of roughly 1.5 × 1.5 cm. (**b**) A vibratome is used to cut 300 μm-thick slices. (**c**) Slices are transferred onto semi-permeable membrane inserts in 6-well culture plates containing slice culture medium. (**d**) hCSF is collected at autopsy from the lateral ventricles using a syringe and spinal needle and slices are cultured with or without hCSF supplementation to the culture medium. (**e**) Timeline of the experimental procedure: slices are prepared and cultured up to 42 DIV. Slices are fixed in PFA once every week and slice-conditioned medium is collected for further analysis

The detailed protocol for obtaining HPMB-OSCs (Fig. 1) can be found in the Supplementary Method (Additional file 1: Supplementary Method). In short, tissue blocks of roughly 1.5 × 1.5 cm were mounted on a Vibratome (Thermo Fisher, Microm HM 650 V) containing ice-cold dissection medium. Slices of 300 μm-thickness were cut perpendicular to the cortical surface. Some slices were immediately fixed in 4% paraformaldehyde (PFA) and used as reference. The remaining slices were transferred onto semi-permeable membrane inserts (Merck-Millipore, PICM0RG50) in six-well culture plates containing 1 ml of normal slice culture medium. Normal slice culture medium consisted of 50% Minimum Essential Medium (Thermo Fisher, 32360026), 25% Earle’s Balanced Salt Solution (Thermo Fisher, 24010043), 25% heat-inactivated horse serum (HIHS; Thermo Fisher, 26050088) supplemented with 1% GlutaMAX-I (Thermo Fisher, 35050038), 1% penicillin-streptomycin, 1.25 µg/ml Amphotericin B (Thermo Fisher, 15290018), and 2.6 mg/ml D-Glucose (45% in dH_2_O; Sigma-Aldrich, G8769).

A proportion of HPMB-OSCs was supplemented with hCSF, obtained from the same donor depending on availability. hCSF was centrifuged for 5 min at 2200 rpm at room temperature (RT) and added in a 1:1 dilution (v/v) to enriched slice culture medium (Additional file 1: Supplementary Method). Enriched slice culture medium contained doubled concentrations of supplements. The remaining hCSF aliquots were stored at -20^o^C.

The slices were cultured up to six weeks at 37°C with 5% CO_2_ in a humidified incubator. VWM patient-derived HPMB-OSCs, the first obtained, were cultured up to three weeks only. Either normal slice culture medium or hCSF supplemented slice culture medium (referred to as –hCSF and + hCSF medium, respectively) was refreshed at 1 day in vitro (DIV) and then three times a week.

### Slice fixation and slice-conditioned medium collection

At 7 DIV, one slice per six-well plate was fixed with 4% PFA. Fixed slices were stored in 0.05% sodium azide in phosphate-buffered saline (PBS) at 4^o^C. Slice-conditioned medium (CM) was collected in sterile Eppendorf tubes and stored at -80^o^C. The remaining slice-containing inserts were transferred to new sterile culture plates and cultured as previously described. These steps were repeated at 14, 21, 28, 35 and 42 DIV.

### Electrophysiological recordings

HPMB slices were visualized on an upright microscope and whole-cell patch-clamp recordings were made using a Multiclamp 700B (Molecular Devices) as previously described [22]. Slices obtained from donor PSY2 and PSY3 were recorded at 7 DIV and PSY4 at 4 DIV. Slices were continuously perfused with oxygenated artificial CSF (aCSF) [22] heated to 34°C and recording electrodes (3–6 MΩ; borosilicate glass capillaries, Harvard Apparatus) were filled with a K-gluconate-based internal solution (K-gluconate 115mM, HEPES 10mM, KCl 4mM, Mg-ATP 4mM, K-Phosphocreatine 10mM, GTP 0.3mM, EGTA 0.2mM, and biocytin 5 mg/ml, pH 7.2 with KOH, and osmolarity 295 mOsm/kg).

Additionally, multi-electrode recordings were made at 11 DIV on slices obtained from CTRL3 and the MS patient. These slices were prepared at 400 μm thickness rather than 300 μm, to include more neuronal layers for recording purposes. Immediately before recording, slices were excised in two, and kept in a chamber with humidified carbogen gas (95% O_2_/5% CO_2_). Slices were then mounted on planar multi-electrode arrays (MED-P5155, 8 × 8, 50 μm-diameter and 5 mm height electrodes interspaced by 150 μm, Alpha MED Sciences, Osaka, Japan) with the electrodes in contact with the bottom side of the slice, perfused with aCSF at a flow rate 1–2 ml/min, and kept submerged at RT in a carbogenated atmosphere. The following solutions were used as perfusate during recordings: (a) aCSF with the following composition in mM: NaCl 125, KCl 3, MgSO4 1, CaCl2 2, Na2 HPO4 1.25 NaHCO3 25–26 and glucose 11, pH 7.4, osmolality ∼300 mOsmol/kg, oxygenated with carbogen; (b) aCSF containing the potassium-channel blocker 4-aminopyridine (4AP, 25 µM; Sigma Aldrich), to induce ictal-like activity; or (c) aCSF containing the sodium-channel blocker tetrodotoxin (TTX, 1 µM, Hellobio). Two consecutive five minute-recordings (Med64 conductor) of spontaneous activity were made upon starting the perfusion of the different types of aCSF. Field potentials were acquired simultaneously at 20 kHz from 64-recording electrodes in the MED64 performer system (Alpha MED Sciences, Osaka, Japan). Analyses were done using custom-made scripts in IGOR Pro 9 software (Wavemetrics, Natick, USA).

### Lysolecithin treatment

At 7 DIV, demyelination was induced by incubating HPMB-OSCs with 0.5 or 1.0 mg/ml lysophosphatidylcholine (LPC; Sigma-Aldrich, L4129) diluted in–hCSF medium. After 18 h incubation, slices were cultured as described and fixed at 11, 14 and 42 DIV.

### AAV vector construction and production

The adeno-associated virus (AAV) transfer plasmid pCBh-eGFP contained a CBh promoter [28] driving enhanced green fluorescent protein (eGFP) as shown in Supplementary Fig. 1 (Additional file 2: Supplementary Fig. 1). The AAV vector prep with capsid PHP.eB [12] was produced, ultracentrifuged and purified through diafiltration at the Viral Vector Facility of the Neuroscience Center Zurich, and kindly provided as a gift from Dr. Gerald Schwank (Department of Pharmacology and Toxicology, University of Zurich, Zurich, Switzerland).

Physical titers (vector genomes/ml) were determined using a Qubit 3.0 Fluorometer (Life Technologies). Identity of the packaged genome of the AAV vector was confirmed by Sanger DNA sequencing Mycrosynth AG (Balgach, Switzerland). The final physical titer of the stock was 8.4 × 10E13 vector genomes/ml and the AAV-PHP.eB-CBh-eGFP vector was stored at -80°C until use.

### Lentivirus production and titration

The third-generation self-inactivating lentiviral vector system with transfer plasmid pCCL_MND_EGFP_bPRE4_SIN, referred to as Lenti-MND-eGFP here, has been described previously [29] and is shown in Supplementary Fig. 2 (Additional file 3: Supplementary Fig. 2). In brief, vector production was performed in adherent HEK293T cells (CRL-11268, ATCC) and cultured in Dulbecco’s modified Eagle’s medium (DMEM + GlutaMAX, 4.5 g/L D-glucose, 110 mg/L sodium pyruvate; Gibco, 10569010) supplemented with 10% (v/v) fetal bovine serum (Gibco, 26140079), 10 units/ml Penicillin and 10 µg/ml Streptomycin (Gibco, 11548876). Cells were grown at 37°C with 5% CO_2_ in a humidified incubator. HEK293T cells were transfected with pCCL_MND_EGFP_bPRE4_SIN, pMDLg/pRRE, pMD-VSV-g and pRSV-Rev using polyethylenimine (Polysciences, 23966). The supernatant containing the lentiviral vectors was harvested 40 h after transfection, cleared from cellular debris by centrifugation at 1,000x*g* for 5 min at RT and then passed through a sterile 0.45 μm pore syringe filter with polyethersulfone membrane (VWR International, 514–1261). The lentiviral vectors were concentrated by centrifugation in an Amicon Ultra-15 centrifugal filter (100kDA cut-off, Merck Millipore, UFC910024) and subsequently in a polyallomer conical centrifuge tube (Seton Scientific, 5022) using ultracentrifugation with a Sorvall Discovery M120 SE centrifuge. A volume of 5 ml per tube was centrifuged in a Sorvall S100-AT6 rotor at 50,000x*g* for 1.5 h at 10°C. Supernatant was removed and viral vector pellets were resuspended in 100 µl PBS overnight at 4°C and stored at -80°C until use. The viral titer was determined by serial dilutions on HEK293T cells measured by flow cytometry (Attune™ NxT Flow Cytometer, Invitrogen) at 72 h post transduction. Final titer was 1 × 10E11 vector genomes/ml.

### Viral transduction

At 4 DIV, 40 µl diluted viral vector in PBS was directly applied to the slice in droplets. The solution covered the whole slice surface while avoiding leakage onto the surrounding membrane insert. Three doses were tested, corresponding to 2 × 10E10, 2 × 10E11 and 2 × 10E12 vector particles for the AAV-PHP.eB serotype and 2.5 × 10E7, 2.5 × 10E8 and 2.5 × 10E9 vector particles for the lentiviral vector. Slices were incubated for 24 h at 37°C and 5% CO_2_, carefully washed, transferred to new culture plates with fresh medium and cultured for an additional 7 days. Slices were fixed at 11 DIV (7 days post-transduction) and vector expression was assessed by GFP immunohistochemistry.

### Viability assays

#### LDH cytotoxicity assay

CyQUANT lactate dehydrogenase (LDH) Cytotoxicity assay (Thermo Fisher, C20301) was performed according to manufacturer’s instructions to assess cell membrane integrity. Fifty µL of slice-CM samples, obtained from slices cultured in either –hCSF or + hCSF medium, was used in triplicate. Pure –hCSF and + hCSF medium controls were included to account for LDH background signal present in serum and hCSF, and data were displayed as percentage of the corresponding medium control.

#### LIVE/DEAD assay

LIVE/DEAD viability assay (Thermo Fisher, L3224) was performed according to the manufacturer’s protocol on a single slice obtained from PSY4. At 42 DIV, the slice was incubated in 1 ml of PBS supplemented with 4µM calcein-AM and 2µM ethidium homodimer-1 for 45 min at RT. The slice was washed twice in PBS and imaged in the culture plate on a Nikon LIPSI microscope using a 10x objective with a z-step size of 5 μm. Live and dead cells were identified by green calcein or red ethidium fluorescence, respectively.

### Histology and imaging

Immunohistochemistry and immunofluorescence were applied on either whole-mount PFA-fixed slices or paraffin-embedded tissue sections of 5 μm-thickness. For free-floating immunostaining of whole-mount slices, slices were first blocked in 3% (v/v) HIHS, 2% (w/v) bovine serum albumin, and 0.5% (v/v) Triton X-100 in PBS. Slices were incubated with primary antibodies in blocking buffer for 96 h at 4^o^C. Sections were then incubated with appropriate Alexa Fluor®-conjugated secondary antibodies (1:250; Thermo Fisher) and counterstained with DAPI (5 µg/mL; Sigma-Aldrich, D9542). Due to the thickness of the whole-mount slices, two 18 mm coverslips were fixed on top of one another at each end of a SuperFrost Plus glass slide (Thermo Fisher) with nail polish. The tissue slice was mounted in between the coverslips using Fluoromount-G (Southern Biotech, 0100-01) and a 24 × 50 mm coverslip.

Paraffin-embedded tissue sections were routinely stained with hematoxylin & eosin (HE) and luxol fast blue with periodic acid Schiff (LFB-PAS). Sections were either incubated in 0.3% (w/v) H_2_O_2_ in dH_2_O for 30 min to block endogenous peroxidase activity for immunohistochemistry, or 0.1% (w/v) glycine in dH_2_O for 10 min to block unreacted aldehydes for immunofluorescence. Heat-induced antigen retrieval was performed in 10mM citrate buffer (pH 6.0) or Tris/EDTA buffer (pH 9.0). Tissue sections were then incubated overnight at RT with primary antibodies. For immunohistochemistry, sections were incubated with anti-rabbit/mouse horseradish peroxidase (poly-HRP)-conjugated immunoglobulins (Dako REAL™ EnVision™ HRP Rabbit/Mouse (ENV), K5007) for 30–60 min. Secondary antibody binding was developed with 3,3’-diaminobenzidine chromogen (1:50; Dako, K5007) and sections were counterstained with hematoxylin and mounted using Quick-D (Klinipath, The Netherlands). For immunofluorescence, sections were incubated with appropriate Alexa Fluor®-conjugated secondary antibodies, counterstained with DAPI and mounted using Fluoromount-G.

The following primary antibodies were used: anti-cleaved caspase-3 (rabbit monoclonal, 1:200, Cell signaling, 9664), anti-CD68 (mouse monoclonal, 1:200, Dako, M0814), anti-GFP (chicken polyclonal, 1:1,000, Aves Lab, GFP-1020), anti-GFAP (chicken polyclonal, 1:1,500, Sigma-Aldrich, AB5541; or rabbit polyclonal, 1:1,000, Dako, Z0334), anti-HuC/D (mouse monoclonal, 1:200, Thermo Fisher, A21271), anti-MAP2 (mouse monoclonal, 1:50, Thermo Fisher, 13-1500), anti-MBP (rat monoclonal, 1:300, BioRad, MCA409S), anti-NFH (chicken polyclonal, 1:10,000, BioLegend, 822601), anti-Olig2 (rabbit polyclonal, 1:500, Millipore, AB9610), anti-PLP (mouse monoclonal, 1:3,000, BioRad, MCA839G), anti-P2RY12 (rabbit polyclonal, 1:500, Anaspec, AS-55042A) and anti-SOX10 (rabbit monoclonal, 1:50, Cell Marque, 383R-16).

Images were acquired using a Leica DM4000B light microscope or Leica DM5000B fluorescence microscope using a 20x objective. A Leica SP8 Confocal microscope (Leica microsystems, Germany) was used to obtain z-stacked images of whole-mount HPMB-OSCs with a z-step size of 1 μm using a 40x oil objective.

### Hyaluronan ELISA

A hyaluronan ELISA (R&D Systems, DHYAL0) was performed according to the manufacturer’s protocol. Slice-CM samples were diluted 1:8 in RD5-18. Fifty µl of diluted sample was used in duplicate. The absorbance was measured within 30 min at 450 nm, with a wavelength correction at 540 nm. A four-parameter logistic curve-fit was applied to interpolate the concentration values of the samples based on the standard curve. Pure–hCSF medium and + hCSF medium controls were included to account for hyaluronan background signal present in serum and hCSF, and data were displayed as percentage of the corresponding medium control.

### Protein and RNA isolation

PFA-fixed HPMB-OSCs were lysed in lysis buffer (11mM Tris pH 7.4, 55mM KAc, 5mM EDTA, 1.65mM MgAc2, 2mM DTT, 1% IGEPAL, 1% sodium deoxycholate and 1x HALT [protease and phosphatase inhibitory cocktail, Thermo Fisher Scientific] in non-DEPC treated water [Ambion]) using a tissue homogenizer (Cole-Parmer, SHM1). Lysates were further processed and protein concentrations were determined using a Quick Start Bradford Protein Assay (Bio-Rad) as previously described [49]. For RNA isolation, 200 µL of lysate supernatant was added to TRIzol Reagent (Invitrogen) and total RNA was isolated as previously described [49]. RNA quality and quantity were determined by calculating the ratio of absorbance measured at 260 and 280 nm (NanoDrop 2000, Thermo Fisher).

### Data analysis

Pictures were analyzed using ImageJ 1.51 W software (NIH, Bethesda, USA). Hematoxylin-stained cell nuclei were manually counted on a minimum of five images per slice using the cell counter plugin. The live/dead cell quantification was performed using the analyze particles plugin and the percentage of live cells relative to the total number of cells was calculated. Data was visualized using Prism 9.3.1 software (GraphPad Software Inc., San Diego, USA). Data are displayed as mean of technical replicates. Due to the inclusion of patients with heterogeneous diagnoses and to assess all donor-derived HPMB-OSCs individually, no data were pooled and no statistical tests were performed.

## Results

### HPMB-OSCs demonstrate long-term viability ex vivo

HPMB-OSC viability was assessed during a six-week culture period. In all slices cultured in–hCSF medium, the degree of cytotoxicity, as measured by the amount of LDH release, was increased relative to the medium control at 7 DIV as shown in Supplementary Fig. 3 (Additional file 4: Supplementary Fig. 3a). On average, LDH release in–hCSF medium was roughly normalized to the culture medium control after two-three weeks in culture. Throughout the rest of the culture period, LDH release remained relatively stable (Additional file 4: Supplementary Fig. 3a), indicating viability up to at least six weeks ex vivo. Cleaved caspase-3 staining at 42 DIV showed negligible apoptosis-related cell death in HPMB-OSCs of multiple donors (Additional file 4: Supplementary Fig. 3b). In addition, the LIVE/DEAD assay in a single slice showed a larger proportion of calcein-AM+ (living) cells relative to ethidium homodimer-1+ (dead) cells at 42 DIV (Additional file 4: Supplementary Fig. 3c). The calculated cell viability across multiple z-stacks averaged 67%. RNA and protein content of HPMB-OSC was also assessed from PFA-fixed cultured slices at 42 DIV. Good-quality RNA and protein was extracted as shown by the 260/280 absorbance ratio and total content presented in Supplementary Table 1 (Additional file 5: Supplementary Table 1). Combined, these data demonstrate long-term viability of HPMB-OSCs ex vivo.

### Supplementing HPMB-OSCs with hCSF may improve slice recovery

For the majority of slice cultures, addition of hCSF reduced LDH release in the first one-two weeks ex vivo as shown in Supplementary Fig. 3 (Additional file 4: Supplementary Fig. 3a). This suggests reduced cytotoxicity following slice preparation upon hCSF supplementation. Inter-donor variations were, however, apparent. In CTRL1, for example, LDH release was higher when cultured with hCSF than without. This could be due to the older age of the donor and/or the composition of the donor’s hCSF. Also in slices supplemented with hCSF, a decreased LDH release after the first one-two weeks ex vivo was observed for all donors, with relatively stable LDH release afterwards (Additional file 4: Supplementary Fig. 3a). This indicates long-term viability of HPMB-OSCs using both culture methods.

### HPMB-OSCs retain tissue structure and diversity of neural cell types

**Fig. 2:**
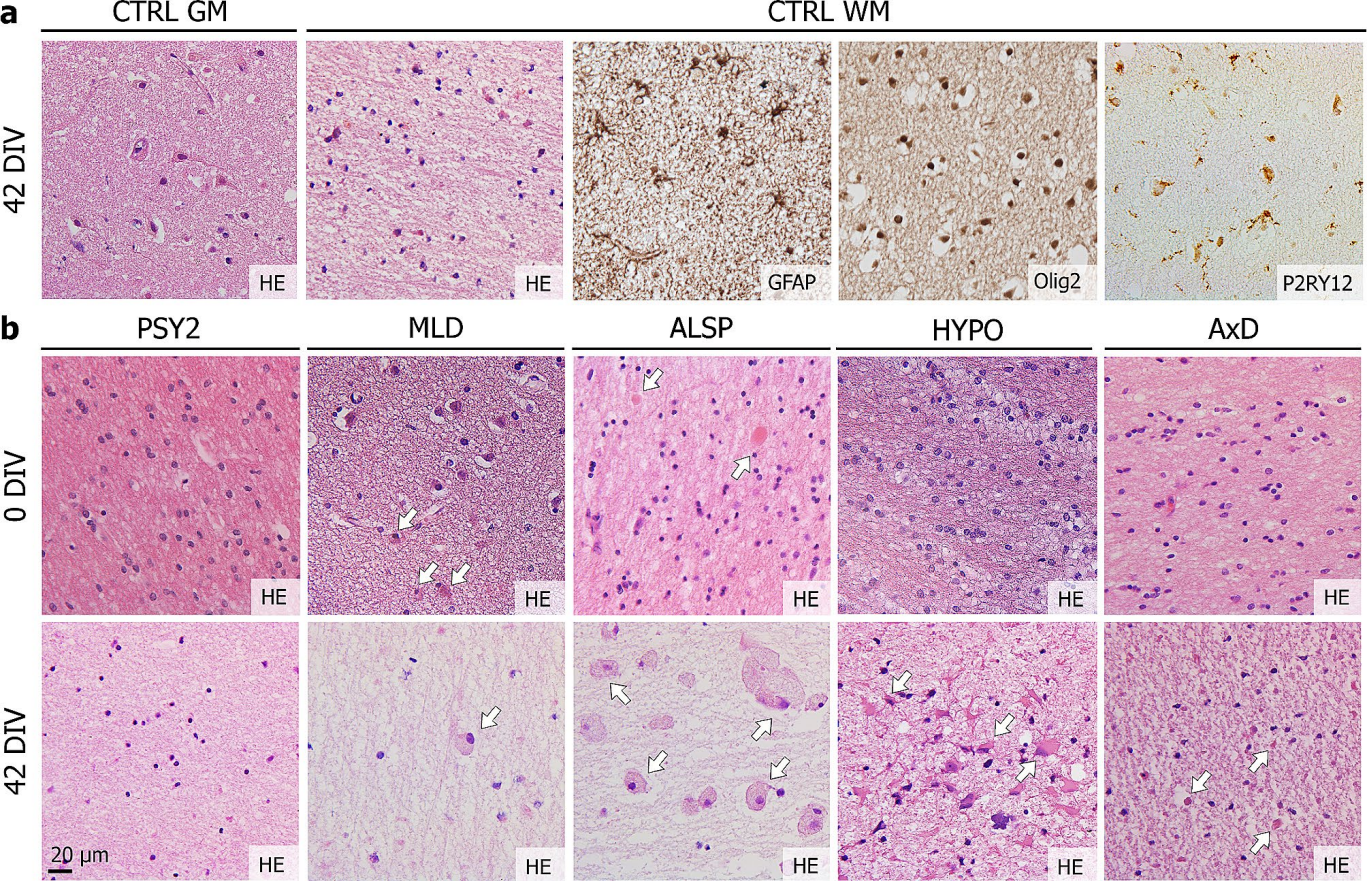
HPMB-OSCs maintain relatively normal tissue structure and diversity of neural cell types. (**a**) Control HPMB-OSCs show preservation of gross tissue structure based on HE staining and presence of neurons in the grey matter (GM) and GFAP+ astrocytes, Olig2+ oligodendrocytes and P2RY12+ microglia in the white matter (WM) at 42 DIV. Expression of P2RY12, a marker for non-activated microglia, together with the ramified morphology, indicates a homeostatic cell state of microglia in HPMB-OSCs even up till 42 DIV. (**b**) HE staining of uncultured reference slices (0 DIV) and slices cultured without hCSF (42 DIV) from multiple donors shows relative preservation of the histo-architecture, but a clear reduction in total cell numbers, after six weeks in culture. Additionally, disease-specific pathology is observed in both reference and cultured slices, as indicated by white arrows. This includes enlarged, rounded microglia/macrophages in MLD patient-derived slices in the white matter and at the subcortical boundary. Axonal spheroids (reference slice) and pigmented glia (cultured slice) are observed in ALSP patient slices and robust reactive astrogliosis in HYPO patient slices. Small Rosenthal fibers are observed in AxD patient slices at 42 DIV

HPMB-OSCs derived from controls demonstrated well-preserved tissue structure and presence of all (neural) cell types (neurons, astrocytes, oligodendrocytes, microglia and endothelial cells) up to at least six weeks in culture (Fig. 2a). To assess the impact of culturing on tissue structure, uncultured reference slices (0 DIV) were compared to cultured slices (42 DIV). Although the total cell density was decreased after six weeks in culture for all donors as shown in Supplementary Fig. 4 (Additional file 6: Supplementary Fig. 4) and Supplementary Fig. 5 (Additional file 7: Supplementary Fig. 5), the brain histo-architecture and disease-specific characteristics were maintained (Fig. 2b). Cell density decreased the most during the first one-two weeks in culture (Additional file 6: Supplementary Fig. 4), in line with LDH viability data (Additional file 4: Supplementary Fig. 3a). HE staining of HPMB slices cultured for 42 DIV in the absence or presence of hCSF showed no differences in tissue structure or cell density for all donors (Additional file 7: Supplementary Fig. 5). These findings confirm that both culture media promote long-term viability of HPMB-OSCs ex vivo. We observed, however, less signs of reactive gliosis in the HYPO patient slices upon supplementing the medium with hCSF than without (Additional file 7: Supplementary Fig. 5a).

### Neurons in HPMB-OSCs remain viable and functional

To assess the electrophysiological properties of neurons in HPMB-OSCs, whole-cell patch-clamp recording was attempted at 4 and 7 DIV on slices obtained from three donors. Numerous pyramidal neurons of normal morphology were present in all slices, however in none a >1GΩ seal could be created. Upon approaching the cells with the recording electrode, the cell membranes appeared stiff and, rather than making a seal with the electrode, the cells were pushed away. The patch-clamp method is therefore not suitable to assess neuronal activity in HPMB-OSCs, at least at the reported time points using the methods described.

**Fig. 3:**
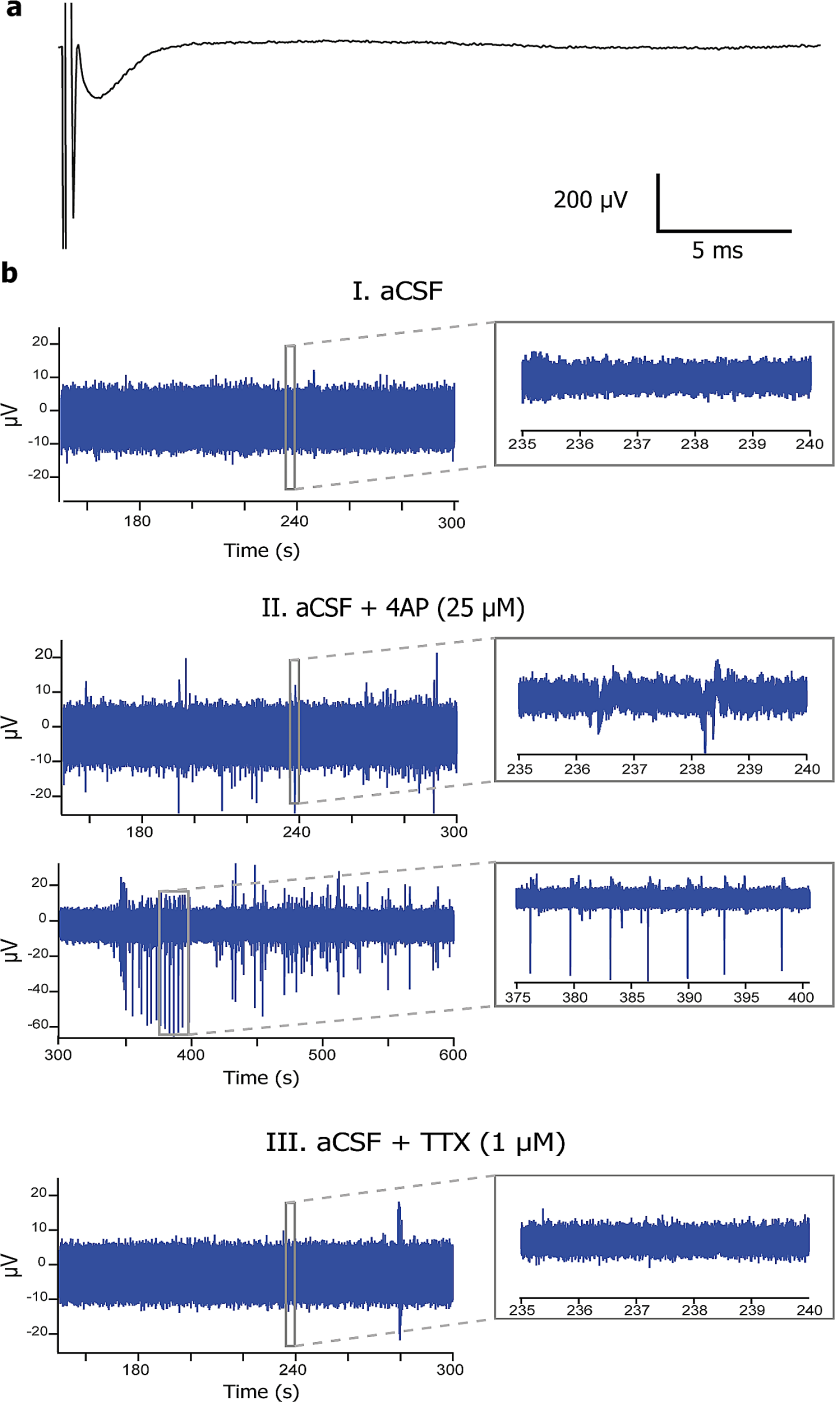
Electrophysiological recordings in HPMB-OSCs demonstrate neuronal activity ex vivo. (**a**) Field potential recorded upon electrical stimulation of a HPMB slice obtained from CTRL3 at 11 DIV shows neuronal activity ex vivo. (**b**) Representative examples of extracellular activity recorded from a HPMB slice obtained from the MS patient at 11 DIV during the incubation with normal aCSF (panel I), aCSF with 25 µM 4AP (panel II), and aCSF with 1 µM TTX (panel III). 4AP increases neuronal activity, whereas TTX abolishes the neuronal firing. Inserts show an expanded view of the recorded events at the time period indicated by the box

Using extracellular multi-unit recordings, by contrast, field potentials were recorded upon electrical stimulation at 11 DIV (Fig. 3a), demonstrating neuronal activity ex vivo. Addition of 4AP blocking voltage-gated potassium channels increased extracellular activity, whereas TTX blocking sodium channels decreased the extracellular activity (Fig. 3b). These data indicate that neurons remain viable and that their ion channel and electrophysiological functions are maintained in HPMB-OSCs obtained from both control and diseased donors.

### Leukodystrophy patient-derived HPMB-OSCs maintain disease-specific characteristics

**Fig. 4:**
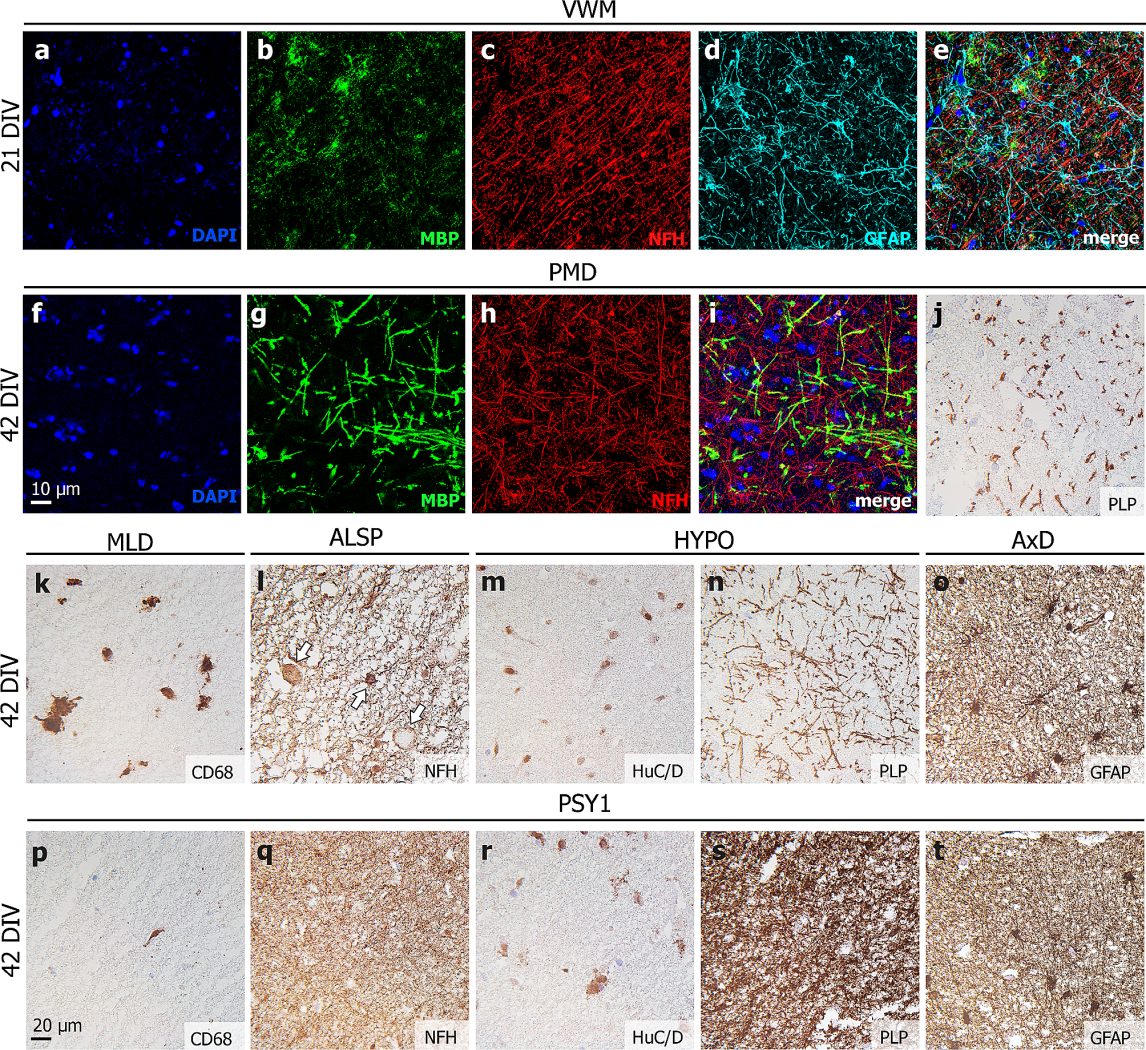
Disease-specific neuropathology in leukodystrophy patient-derived HPMB-OSCs. (**a**-**e**) Free-floating staining of whole-mount slices of the VWM patient at 21 DIV shows paucity of MBP+ myelin, relative preservation of NFH+ axons and dysmorphic GFAP+ astrocytes. (**f**-**i**) Free-floating staining of whole-mount PMD patient-derived slices at 42 DIV shows a lack of MBP+ myelin, (**j**) which is also appreciated when staining paraffin-embedded sections for PLP. (**k**) In MLD patient slices, CD68+ microglia/macrophages show increased lysosomal activity. (**l**) ALSP patient-derived HPMB-OSC demonstrates NFH+ axonal spheroids of different sizes (white arrows). (**m**) In HYPO patient slices, altered staining patterns for neuronal marker HuC/D and (**n**) hypomyelination on PLP staining can be appreciated. (**o**) AxD patient slices show dysmorphic, double-nucleated GFAP+ astrocytes. (**p**-**t**) None of the described neuropathological features are observed in HPMB-OSCs derived from control or psychiatric donors

Throughout the culture period, leukodystrophy patient-derived OSCs retained disease-specific characteristics (Fig. 4). At 21 DIV, VWM slices showed paucity of MBP+ myelin, relative preservation of NFH+ axons and dysmorphic GFAP+ astrocytes (Fig. 4a-e), corresponding to described VWM pathology [9]. At 42 DIV, PMD slices showed hypomyelination, as indicated by the lack of MBP+ myelin surrounding NFH+ axons (Fig. 4f-i) and by lack of PLP staining (Fig. 4j); hypomyelination is the hallmark of this disease [25]. MLD slices harbored CD68+ microglia/macrophages (Fig. 4k). As increased CD68 expression indicates phagocytic activity, these microglia engulfed myelin debris and/or accumulated sulfatides, as typically seen in MLD [50]. ALSP slices showed axonal spheroids of different sizes indicating axonal degeneration (Fig. 4l). Spheroids are focal axonal swellings containing organelles and disrupted cytoskeletal filaments [51]. This is a pathological feature to which the disease ALSP owes its name [1, 36]. Slices obtained from the HYPO patient demonstrated relative absence of cortical pyramidal neurons as seen with the neuronal marker HuC/D (Fig. 4m) and hypomyelination on PLP staining (Fig. 4n). These features are compatible with hypomyelination secondary to a cortical disorder. AxD slices showed dysmorphic GFAP+ astrocytes (Fig. 4o), also double-nucleated, congruent with literature [32]. All mentioned neuropathological features were not observed in HPMB-OSCs derived from control or psychiatric donors (Fig. 4p-t).

**Fig. 5:**
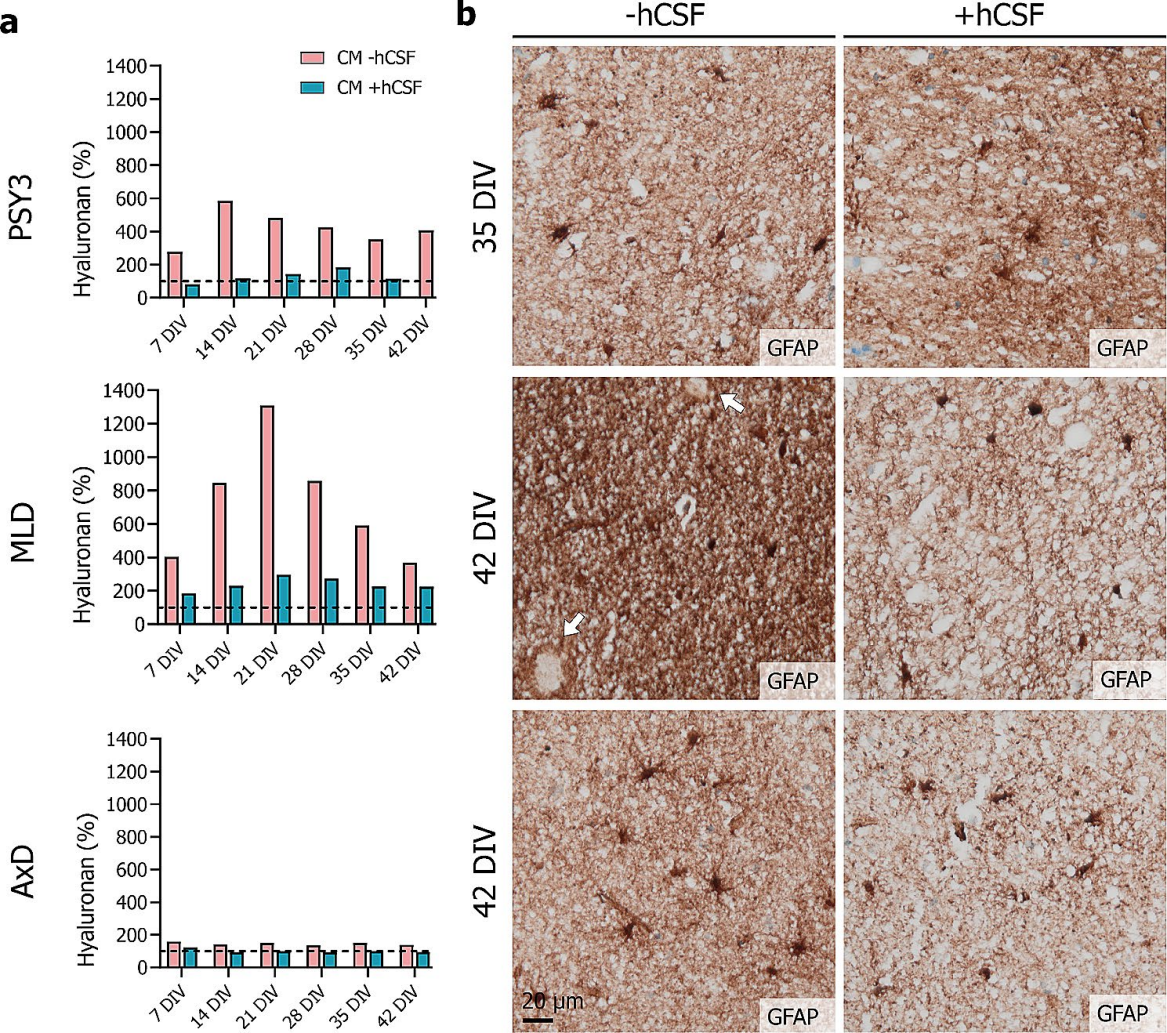
Hyaluronan levels in slice-CM of HPMB-OSCs. (**a**) Hyaluronan levels determined by ELISA in slice-CM of HPMB-OSCs obtained from PSY3, the MLD patient, and the AxD patient (from top to bottom) cultured without (pink) or with (blue) addition of hCSF. The dotted line represents the–hCSF and + hCSF medium controls, set at 100%. Hyaluronan levels are presented as percentage of the corresponding medium control. Data are displayed as mean of technical replicates and hence no statistical analysis could be performed. (**b**) GFAP staining of slices cultured without (left) or with (right) hCSF obtained from PSY3, the MLD patient and AxD patient (from top to bottom), showing decreased immunoreactivity for GFAP in slices supplemented with hCSF

To assess factor secretion in slice-CM, hyaluronan levels were determined using sandwich ELISA. For all cultures, hyaluronan concentrations above culture medium control levels were detected throughout the culture period, indicating that cells in HPMB-OSCs secrete hyaluronan and hence remain viable up till six weeks ex vivo (Fig. 5a). In psychiatric donor-derived HPMB-OSCs, hyaluronan levels were increased at all time-points, while supplementing with hCSF decreased hyaluronan levels to medium control levels (Fig. 5a). Unexpectedly, MLD slices behaved differently. Culturing without hCSF markedly increased hyaluronan levels compared to control, and levels only partly reduced upon hCSF supplementation (Fig. 5a). In AxD slices, only subtle changes in hyaluronan levels were detected throughout the culture period, remaining overall comparable to culture medium control levels in both conditions (Fig. 5a). The changes in hyaluronan levels in slice-CM from HPMB-OSCs upon hCSF supplementation co-varied with the immunoreactivity for GFAP (Fig. 5b). Hyaluronan is mainly secreted by reactive astrocytes. This suggests that adding hCSF restricts the post-slicing reactive gliosis. This is also in line with the observation of meager reactive gliosis in the HYPO patient slices upon supplementing the medium with hCSF (Additional file 7: Supplementary Fig. 5a).

### HPMB-OSCs respond to injury ex vivo

**Fig. 6:**
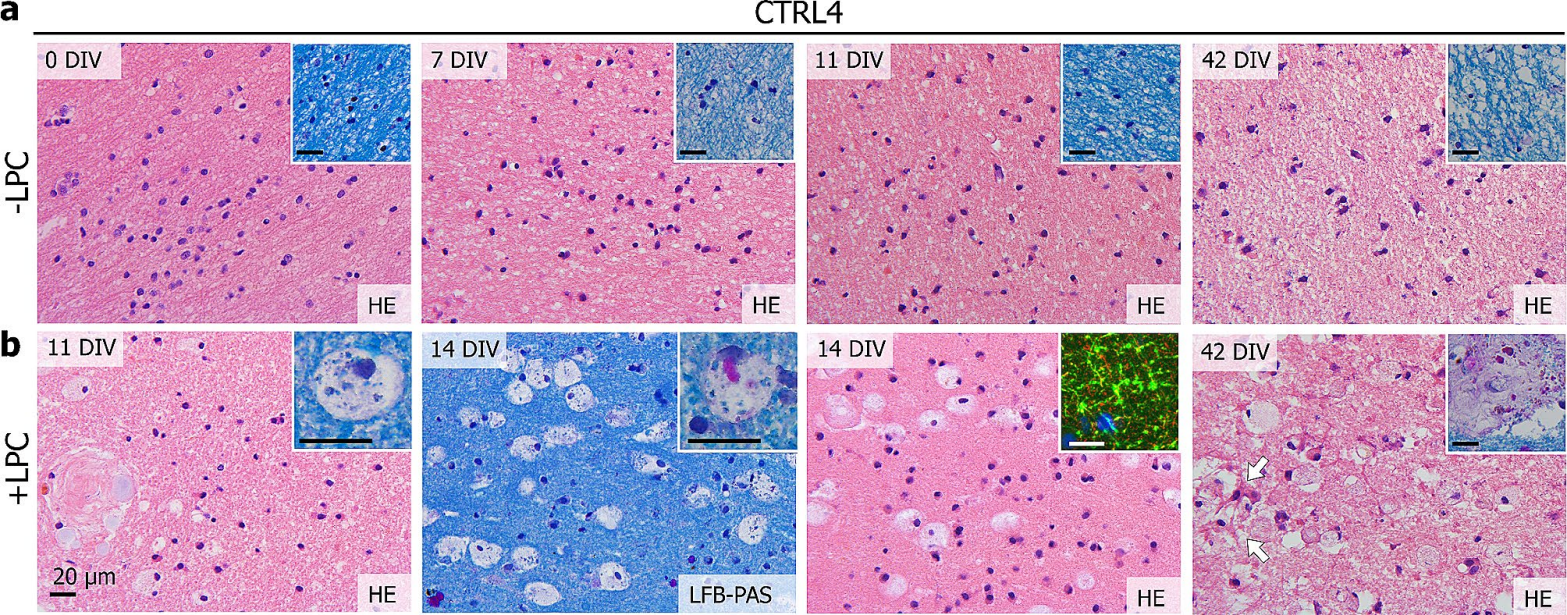
Lysolecithin treatment of HPMB-OSCs induces a multicellular response to injury ex vivo. (**a**) HPMB-OSCs obtained from CTRL4 that are not treated with LPC show normal tissue structure and cell morphology based on HE and LFB-PAS staining (inserts) at 0 DIV (uncultured reference slices) and 7, 11 and 42 DIV. (**b**) Slices treated with LPC show many rounded microglia/macrophages at 11, 14 and 42 DIV. The microglia/macrophages have an enlarged cytoplasm and show LFB+ vacuoles (11 DIV insert) and sometimes PAS+ vacuoles (14 DIV insert). At 14 DIV, myelin swelling is observed based on MBP (green) and NFH (red) staining (insert). At 42 DIV, rounded macrophages are still present, as well as signs of reactive astrogliosis (white arrows). The macrophages are also found close to the blood vessels and even inside the vascular adventitia (42 DIV insert). Data obtained using 1.0 mg/ml LPC are shown. A similar but milder response was obtained using 0.5 mg/ml LPC. All scale bars represent 20 μm

To examine if HPMB-OSCs model intrinsic repair upon injury, slices were treated at 7 DIV with LPC to induce demyelination. A physiological microglial response was observed at 11 and 14 DIV, which persisted up to at least 42 DIV (Fig. 6). Microglia assumed a macrophage morphology with rounded, enlarged bodies and foamy cytoplasm filled with LFB+ or PAS+ vacuoles (Fig. 6b). This finding indicates that damage of myelin upon LPC treatment is cleared by macrophages engulfing myelin debris. At 14 DIV, myelin swelling was observed on MBP-NFH stained slices (Fig. 6b). At 42 DIV, macrophages were still present, together with evidence of reactive astrogliosis. Macrophages were found around blood vessels and even inside the vascular adventitia (Fig. 6b), suggesting that macrophages can indeed migrate in the slices.

### HPMB-OSCs are suitable to test gene therapy vectors ex vivo

**Fig. 7:**
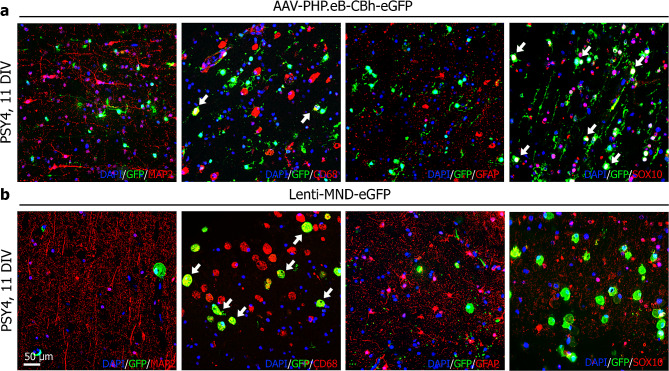
Successful viral transduction of HPMB-OSCs. (**a**) In HPMB-OSCs derived from PSY4 transduced with the AAV-PHP.eB-CBh-eGFP viral vector, predominant colocalization of GFP with SOX10 and CD68 is found at 11 DIV, but no colocalization with MAP2 or GFAP. (**b**) Using the Lenti-MND-eGFP vector, colocalization of GFP with CD68 is observed, but not with MAP2, GFAP or SOX10. Data of the highest of three doses of both viral vectors is shown. Similar results were obtained for lower doses in a concentration-dependent manner

To assess if viral transduction of HPMB-OSC ex vivo is possible, slices were treated with either an AAV or lentiviral vector at 4 DIV. Successful transduction of both viral vectors was obtained, as indicated by the abundant presence of GFP+ cells at 11 DIV (Fig. 7). Using the AAV-PHP.eB serotype, predominant colocalization of GFP with SOX10 (oligodendrocytes) and CD68 (microglia and macrophages) was observed in the white matter (Fig. 7a). Lentiviral transduction mainly showed colocalization of GFP with CD68 in the white matter (Fig. 7b). In neither case did we observe colocalization of GFP with GFAP+ (astrocytes) or MAP2+ cells (neurons) (Fig. 7).

## Discussion

Here, we describe an adapted ex vivo OSC method using HPMB tissue obtained from patients with leukodystrophies, psychiatric disorders, and control donors. This method guarantees prolonged viability, tissue integrity and 3D histo-architecture, and preservation of all (neural) cell types. Fundamentally, patient-derived OSCs maintain disease-specific hallmarks throughout the culture period. The method is therefore suitable for studying all neural cells in healthy and both morphologic and functional disease conditions, and can be used as a tool to study disease mechanisms and therapy development.

Viability of HPMB-OSCs was assessed during a six-week culture period. In all slice cultures, the degree of cytotoxicity as indicated by the degree of LDH release in slice-CM is increased at one week after slice preparation relative to control. This can be attributed to cutting of the tissue block. Slice preparation inevitably damages some cells at the surface, as also confirmed by the decrease in total cell numbers after one week. In agreement with this, cultured slices develop reactive astrogliosis at the cutting edges, also indicating viability and preserved response to injury. On average, the degree of cytotoxicity normalized to the culture medium control by two weeks ex vivo suggesting that, by this time point, tissue slices recover from injury and that cells are viable. Between two and six weeks ex vivo, the degree of cytotoxicity remained relatively stable, implying that HPMB-OSCs remain viable during this prolonged culture period. These findings are supported by the total cell nuclei counts and the LIVE/DEAD assay performed at six weeks ex vivo.

hCSF supplementation to the culture medium can improve tissue recovery of HPMB-OSCs after slice preparation. hCSF restricts the degree of cytotoxicity in the first one-two weeks following slice preparation. Few studies have previously addressed this possibility; improved neuron viability and maintenance of network activity in human OSCs obtained at surgery was described when using hCSF from hydrocephalus patients [39]. In the present study, hCSF and tissue specimens were derived from the same donor to more closely mimic the donor’s (micro)environment in vivo. We found, however, no significant differences in tissue structure, total cell numbers, and viability of slices cultured in absence or presence of hCSF even at 6 weeks ex vivo, suggesting that both culture methods support long-term slice survival.

The described HPMB-OSC method offers various experimental applications to study disease mechanisms. As a proof-of-principle, we showed that it is feasible to extract good-quality RNA and protein even from PFA-fixed cultured slices. HPMB-OSCs are therefore suitable for multi-omics approaches, also aiming at establishing the molecular effects of treatments. Isolation of RNA and protein using optimized protocols for PFA-fixed tissue or from snap-frozen rather than PFA-fixed slices, is likely to increase both quantity and quality.

Slice-CM of HPMB-OSCs can be used to assess secreted factors, as shown for extracellular matrix component hyaluronan. Hyaluronan is mainly produced by astrocytes [40] and accumulates in demyelinated lesions, where it hampers oligodendrocyte progenitor maturation [4, 42]. In VWM, hyaluronan is overabundant in the white matter, correlating with the arrested oligodendrocyte progenitor maturation [7, 9, 15]. Except for VWM, hyaluronan metabolism has not been investigated in leukodystrophies. We found donor-specific variation in hyaluronan levels in slice-CM from a psychiatric, MLD and AxD donor. These results strongly suggest that hyaluronan production might be altered in these leukodystrophies and require further studies. In all cultures, hyaluronan levels were strikingly decreased in CM of slices cultured with addition of hCSF compared to those without. Additionally, hyaluronan levels in slice-CM co-varied with GFAP immunoreactivity. This suggests that adding hCSF might limit the post-slicing reactive gliosis. This is in line with our LDH assay data pointing to a beneficial effect of hCSF on cell viability.

We also assessed if HPMB-OSCs are suitable to study neuronal activity. We were unable to record pyramidal neurons at 4 and 7 DIV in HPMB-OSCs obtained from three donors using the patch-clamp technique. The negative results obtained compared to OSCs from surgically-derived tissue [2, 18, 39, 45] might be due to increased time before tissue processing, including post-mortem delay, agonal state at death, and stiffness of the cell membrane, which might relate to hypoxia-induced actin reorganization [11]. Recording from pyramidal neurons in acute rather than cultured HPMB slices is already considered challenging, but not impossible [26]. Recordings in brain tissue within three hours post-mortem revealed typical action potential profiles and synaptic transmission as in acute surgically resected human brain tissue [26]. We do not exclude the possibility that successful patch-clamp recordings can also be achieved in our HPMB-OSC model. Pharmacological or molecular manipulation of the cytoskeleton might resolve the cell membrane stiffness and enable patch-clamp recordings. Using extracellular multi-unit recordings, by contrast, we clearly observed field potentials and extracellular activity in HPMB-OSCs at almost two weeks ex vivo. This indicates that, both in control and diseased tissue, neurons remain viable and functional and that the developed HPMB-OSCs allow for neurophysiology applications.

HPMB-OSCs also respond to injury ex vivo. Even at six weeks ex vivo, control slices harbor microglia of ramified morphology that express P2RY12, a protein associated with homeostasis or non-activated microglia [5, 10]. This demonstrates a resting state of the microglia in response to their microenvironment. Upon LPC treatment to induce demyelination, a physiological multicellular response persists for several weeks ex vivo. We found evidence of reactive astrogliosis, myelin swelling, and foamy macrophages engulfing myelin debris. Clearance of myelin debris is a crucial prerequisite for remyelination in vivo [33, 41]. Whether the myelin swelling in this model represents blebbing or blister formation in patients, has to be determined. The macrophages were found close to the blood vessels and even inside the vascular adventitia, suggesting that these cells can indeed migrate inside the slice. These findings overlap with what happens in response to demyelination in vivo and indicate that HPMB-OSCs can be used as a model to assess intrinsic repair and multicellular responses to injury.

Suitable models for testing capsid engineered AAV variants for translation of gene therapy applications to humans are lacking [20, 31]. Hence, in vitro models as brain organoids are being exploited [14]. Here, we show that ex vivo HPMB-OSCs are applicable to test different types of gene therapy vectors. Successful AAV and lentiviral vector transduction was achieved, as indicated by the abundant presence of GFP+ cells. Using the AAV-PHP.eB serotype, predominant colocalization of GFP with SOX10 and CD68 was observed, indicating that in human tissue this vector targets oligodendrocytes and microglia/macrophages. Lentiviral transduction mainly showed colocalization of GFP with CD68, demonstrating effective transduction of microglia/macrophages only. In neither case did we observe colocalization of GFP with GFAP+ astrocytes or MAP2+ neurons. HPMB-OSCs can thus provide useful information related to transduction efficiency and targeting of specific cell types in human tissue ex vivo. This has important implications for bridging preclinical tests in mice to patients, as it cannot be *a priori* excluded that the cell types transduced by different vectors may differ across species. To further assess the transduction efficiency for the viral vectors tested, these experiments should be replicated on HPMB-OSCs obtained from multiple donors. The successful viral transduction obtained indicates that HPMB-OSCs may be useful for assessment of potential therapies such as gene therapy for brain disorders, including the leukodystrophies. While not addressed in the current study, previously reported use of human brain slices to test therapies [30, 35] suggests that these HPMB-OSCs also allow for testing other regenerative strategies. These include screening pharmacological compounds and culturing exogenous cells onto the slices to assess cell replacement strategies.

The only previously reported studies on OSC obtained from HPMB tissue [30, 34, 35, 48] are based on a method developed by Swaab et al. [47]. In this method, only a part of the sulcus-facing cortex and a thin strip of the underlying white matter was dissected, resulting in relatively small slices, consisting of mostly grey matter. The slices of 200 μm thickness were cut using a Tissue chopper and were cultured free-floating in a 24-well plate [47]. This harvesting method implies a large degree of axotomy, which may impact on neuronal viability and their networks [21]. Additionally, this method includes limited white matter, making the model unsuitable to study white matter disorders like the leukodystrophies. We have adapted the ex vivo OSC method for culturing HPMB tissue by applying the well-established air-liquid interface method using semi-permeable membrane inserts in 6-well plates [43]. In the current method, slices include the entire gyrus with a substantial part of the subcortical white matter to better preserve the integrity of the U-fibers and contain longer projection axons, and are thus also much larger in size. Slices were cut at 300 μm thickness using a Vibratome and cultured using a specific composition of culture medium. Our currently developed HPMB-OSC method better allows for studying live cells in the white matter and is thus more suitable to study white matter disorders.

Limitations include the scarce availability of fresh HPMB tissue from (relatively young) controls and patients with rare disorders as the leukodystrophies [9]. We were therefore unable to include more leukodystrophy patients with similar diagnoses and chose to also include donors with psychiatric disorders. A lack of morphologic neuropathology in the slices of the psychiatric donors does, however, not exclude functional and/or biochemical differences compared to those of non-psychiatric donors. It should therefore be considered to exclude donors with psychiatric disorders in future studies using HPMB-OSCs with the aim to study healthy versus morphologically diseased brain and include larger groups and age-matched controls. The reproducible viability of all donor-derived HPMB-OSCs and the observation of well-described neuropathological characteristics in those of patients for all leukodystrophies studied imply, however, robustness of the technique. Slices from both healthy and functionally or morphologically diseased brains can be used as an ex vivo human brain model. It should also be noted that part of the donors died of legal euthanasia, which may cause differences compared to donors that died a natural death. Such differences have been shown for the hypothalamus [53], but remain unexplored for other brain areas. Additionally, the pH of the hCSF was consistently higher in leukodystrophy than in psychiatric and control donors. This is likely due to the technical reason that pH determination in these patients was done after freeze-thawing cycles, which may increase the pH [44]. We did, however, not observe differences in slice viability with respect to cause of death or pH of the hCSF. Another intrinsic limitation is the lack of an adaptive immune response, as blood does not circulate in the resected vessels. This could be overruled by adding peripheral inflammatory cells to the slices. Additionally, the quality of HPMB tissue and hCSF is influenced by agonal state at death, post-mortem delay and stability during tissue processing. Considering this, we chose to selectively include donors with a post-mortem delay of maximum 6 h to ensure high quality and viability.

These HPMB-OSCs show important advantages over animal models, iPSC-derived models including organoids, and even brain slice cultures derived from surgical resections. HPMB-OSCs are human-derived, lacking inter-species differences; they retain the 3D histo-architecture of the human brain, which is an obvious advantage over other in vitro models; and they are obtainable at any age (as shown by the age of our donors, ranging from 3 to 94 years) and potentially from any brain region, avoiding the developmental fingerprinting of iPSCs and organoids and the limited regional availability of surgically-obtained slices.

## Conclusions

These adapted HPMB-OSCs represent an ex vivo technique suitable for studying disease mechanisms and pathology, as demonstrated by the recapitulation of known disease hallmarks during a prolonged culture period. Hence, this model may be useful for experimental purposes, such as neuronal recordings, and pre-clinical treatment development purposes, including gene therapy. This technique is expected to be applicable to virtually all brain diseases, including tumors, neurodegenerative, psychiatric, developmental and neuroinflammatory diseases, because the human-specific intricate interplay between complex biological mechanisms both within and between cells is essential to study diseases of the human brain.

### Electronic supplementary material

Below is the link to the electronic supplementary material.

**Additional file 1: Supplementary Method.** Human post-mortem organotypic brain slice cultures. Detailed protocol for generating human post-mortem organotypic brain slice cultures.

**Additional file 2: Supplementary Fig. 1.** Schematic of the adeno-associated viral vector pCBh-eGFP. ITR = Inverted terminal repeat, CBh = CMV enhancer/chicken β-actin hybrid promoter, eGFP = enhanced green fluorescent protein, WPRE = Woodchuck Hepatitis Virus (WHV) posttranscriptional regulatory element, bGH polyA = bovine growth hormone polyadenylation signal.

**Additional file 3: Supplementary Fig. 2.** Schematic of the lentiviral vector pCCL_MND_EGFP_bPRE4_ SIN. LTR = Long-terminal repeat, psi sequence (Ψ) = RNA packaging signal, cPPT = central purine tract, GAG = HIV-1 GAG protein, RRE = Rev responsive element, MND = murine leukemia virus-derived promoter, eGFP = enhanced green fluorescent protein, WPRE = Woodchuck Hepatitis Virus (WHV) posttranscriptional regulatory element.

**Additional file 4: Supplementary Fig. 3.** Long-term viability of HPMB-OSCs ex vivo. (**a**) LDH release in slice-CM of HPMB-OSCs obtained from donors CTRL1, PSY3, MLD, ALSP cultured without (pink) or with (blue) addition of hCSF. The dotted line represents the–hCSF and + hCSF medium controls, set at 100%. LDH release is presented as percentage of the corresponding medium control. Data are displayed as mean of technical replicates and hence no statistical analysis was performed. (**b**) Cleaved caspase-3 (CASP3) staining shows limited signs of apoptosis-related cell death at 42 DIV in patient and control slices in both the grey matter (GM) and white matter (WM). (**c**) LIVE/DEAD assay of HPMB-OSC obtained from PSY4 at 42 DIV with calcein-AM visualizing living cells and ethidium homodimer-1 (EthD-1) indicating dead cells.

**Additional file 5: Supplementary Table 1.** RNA and protein content of PFA-fixed HPMB-OSCs at 42 DIV.

**Additional file 6: Supplementary Fig. 4.** Cell density in control HPMB-OSC without hCSF supplementation throughout the culture period. (**a**) HE staining of HPMB-OSC of PSY2 (top) and PSY4 (bottom) cultured without hCSF supplementation at 0, 7, 14, 35, and 42 DIV shows a decrease in total cell number with increasing time in culture. Although the cell density is decreased after six weeks in culture, there is a considerable number of viable cells and the overall tissue structure remains well preserved. (**b**) Density of hematoxylin-stained cell nuclei in uncultured slices (0 DIV) and slices cultured up till 42 DIV without hCSF of PSY2 (left) and PSY4 (right). In both tissue cultures, the decrease in cell density is proportionally the largest in the first one to two weeks in culture, after which it relatively stabilizes. Data are displayed as mean of technical replicates and hence no statistical analysis was performed.

**Additional file 7: Supplementary Fig. 5.** Cell density in control and patient-derived HPMB-OSC with and without hCSF supplementation. (**a**) HE staining of the white matter of control and patient-derived HPMB slices cultured without (top) and with (bottom) addition of hCSF at 42 DIV (and 21 DIV in the case of VWM) shows comparable total cell numbers. Disease-specific pathology is observed in slices cultured in both medium types, as indicated by white arrows. VWM patient slices show markedly increased cell density in the more preserved white matter close to the cortex (whole image) and paucity of cells and tissue rarefaction deeper in the white matter (insert) compared to control tissue. Both features are typically seen in VWM. MLD patient slices show enlarged, rounded microglia/macrophages, whereas ALSP patient slices show axonal spheroids and pigmented glia. Robust reactive astrogliosis is observed in HYPO patient slices cultured without hCSF, while addition of hCSF restricts the astrocytic response. (**b**) Density of hematoxylin-stained cell nuclei in control and patient-derived uncultured reference slices (beige) and slices cultured without (pink) and with (blue) hCSF for 42 DIV (and 21 DIV in the case of VWM). No reference slices were available for the VWM and PMD patient and no + hCSF data at 42 DIV was available for some donors due to small hCSF volumes obtained. The total cell density is decreased to similar levels in slices cultured with and without hCSF for 42 DIV. Data are displayed as mean of technical replicates and hence no statistical analysis was performed.

## Data Availability

The datasets used and/or analysed during the current study are available from the corresponding author upon reasonable request.

## Abbreviations

4AP: 4-aminopyridine
3D: three-dimensional
ALSP: adult-onset leukoencephalopathy with axonal spheroids and pigmented glia
AxD: Alexander disease
CM: conditioned medium
CNS: central nervous system
DIV: day in vitro
eGFP: enhanced green fluorescent protein
hCSF: human cerebrospinal fluid
HPMB: human post-mortem brain
HYPO: hypomyelination due to a *TUBB2A* defect
iPSCs: induced pluripotent stem cells
PBS: phosphate-buffered saline
OSC: organotypic brain slice culture
MLD: metachromatic leukodystrophy
MS: multiple sclerosis
PFA: paraformaldehyde
PMD: Pelizaeus-Merzbacher disease
PSY: psychiatric disorder
RT: room temperature
TTX: tetrodotoxin
VWM: vanishing white matter

## References

[1] Adams SJ, Kirk A, Auer RN (2018). Adult-onset leukoencephalopathy with axonal spheroids and pigmented glia (ALSP): integrating the literature on hereditary diffuse leukoencephalopathy with spheroids (HDLS) and pigmentary orthochromatic leukodystrophy (POLD). J Clin Neurosci.

[2] Andersson M, Avaliani N, Svensson A, Wickham J, Pinborg LH, Jespersen B (2016). Optogenetic control of human neurons in organotypic brain cultures. Sci Rep.

[3] Avossa D, Grandolfo M, Mazzarol F, Zatta M, Ballerini L (2006). Early signs of motoneuron vulnerability in a disease model system: characterization of transverse slice cultures of spinal cord isolated from embryonic ALS mice. Neuroscience.

[4] Back SA, Tuohy TM, Chen H, Wallingford N, Craig A, Struve J (2005). Hyaluronan accumulates in demyelinated lesions and inhibits oligodendrocyte progenitor maturation. Nat Med.

[5] Beaino W, Janssen B, Kooij G, van der Pol SMA, van Het Hof B, van Horssen J (2017). Purinergic receptors P2Y12R and P2 × 7R: potential targets for PET imaging of microglia phenotypes in multiple sclerosis. J Neuroinflammation.

[6] Birgbauer E, Rao TS, Webb M (2004). Lysolecithin induces demyelination in vitro in a cerebellar slice culture system. J Neurosci Res.

[7] Bugiani M, Postma N, Polder E, Dieleman N, Scheffer PG, Sim FJ (2013). Hyaluronan accumulation and arrested oligodendrocyte progenitor maturation in vanishing white matter disease. Brain.

[8] Bugiani M, Dubey M, Breur M, Postma NL, Dekker MP, Ter Braak T (2017). Megalencephalic leukoencephalopathy with cysts: the Glialcam-null mouse model. Ann Clin Transl Neurol.

[9] Bugiani M, Vuong C, Breur M, van der Knaap MS (2018). Vanishing white matter: a leukodystrophy due to astrocytic dysfunction. Brain Pathol.

[10] Butovsky O, Jedrychowski MP, Moore CS, Cialic R, Lanser AJ, Gabriely G (2014). Identification of a unique TGF-beta-dependent molecular and functional signature in microglia. Nat Neurosci.

[11] Calabrese B, Jones SL, Shiraishi-Yamaguchi Y, Lingelbach M, Manor U, Svitkina TM (2022). INF2-mediated actin filament reorganization confers intrinsic resilience to neuronal ischemic injury. Nat Commun.

[12] Chan KY, Jang MJ, Yoo BB, Greenbaum A, Ravi N, Wu WL (2017). Engineered AAVs for efficient noninvasive gene delivery to the central and peripheral nervous systems. Nat Neurosci.

[13] Chang CY, Ting HC, Liu CA, Su HL, Chiou TW, Lin SZ (2020). Induced Pluripotent Stem cell (iPSC)-Based neurodegenerative disease models for phenotype recapitulation and drug screening. Molecules.

[14] Depla JA, Sogorb-Gonzalez M, Mulder LA, Heine VM, Konstantinova P, van Deventer SJ (2020). Cerebral organoids: a human model for AAV Capsid selection and therapeutic transgene efficacy in the brain. Mol Ther Methods Clin Dev.

[15] Dooves S, Bugiani M, Postma NL, Polder E, Land N, Horan ST (2016). Astrocytes are central in the pathomechanisms of vanishing white matter. J Clin Invest.

[16] Doss MX, Sachinidis A (2019). Current challenges of iPSC-Based Disease modeling and therapeutic implications. Cells.

[17] Dubey M, Bugiani M, Ridder MC, Postma NL, Brouwers E, Polder E (2015). Mice with megalencephalic leukoencephalopathy with cysts: a developmental angle. Ann Neurol.

[18] Eugene E, Cluzeaud F, Cifuentes-Diaz C, Fricker D, Le Duigou C, Clemenceau S (2014). An organotypic brain slice preparation from adult patients with temporal lobe epilepsy. J Neurosci Methods.

[19] Garcia LM, Hacker JL, Sase S, Adang L, Almad A (2020). Glial cells in the driver seat of leukodystrophy pathogenesis. Neurobiol Dis.

[20] Hordeaux J, Wang Q, Katz N, Buza EL, Bell P, Wilson JM (2018). The Neurotropic properties of AAV-PHP.B are limited to C57BL/6J mice. Mol Ther.

[21] Humpel C (2015). Organotypic brain slice cultures: a review. Neuroscience.

[22] Hunt S, Leibner Y, Mertens EJ, Barros-Zulaica N, Kanari L, Heistek TS (2022). Strong and reliable synaptic communication between pyramidal neurons in adult human cerebral cortex. Cereb Cortex.

[23] Jang S, Kim H, Kim HJ, Lee SK, Kim EW, Namkoong K (2018). Long-term culture of Organotypic hippocampal slice from old 3xTg-AD mouse: an ex vivo model of Alzheimer’s Disease. Psychiatry Investig.

[24] Kim H, Kim E, Park M, Lee E, Namkoong K (2013). Organotypic hippocampal slice culture from the adult mouse brain: a versatile tool for translational neuropsychopharmacology. Prog Neuropsychopharmacol Biol Psychiatry.

[25] Koeppen AH, Robitaille Y (2002). Pelizaeus-Merzbacher disease. J Neuropathol Exp Neurol.

[26] Kramvis I, Mansvelder HD, Meredith RM (2018). Neuronal life after death: electrophysiologic recordings from neurons in adult human brain tissue obtained through surgical resection or postmortem. Handb Clin Neurol.

[27] Leferink PS, Dooves S, Hillen AEJ, Watanabe K, Jacobs G, Gasparotto L (2019). Astrocyte subtype vulnerability in Stem Cell models of Vanishing White Matter. Ann Neurol.

[28] Levy JM, Yeh WH, Pendse N, Davis JR, Hennessey E, Butcher R (2020). Cytosine and Adenine base editing of the brain, liver, retina, heart and skeletal muscle of mice via adeno-associated viruses. Nat Biomed Eng.

[29] Lo Presti V, Cornel AM, Plantinga M, Dunnebach E, Kuball J, Boelens JJ (2021). Efficient lentiviral transduction method to gene modify cord blood CD8(+) T cells for cancer therapy applications. Mol Ther Methods Clin Dev.

[30] Luchetti S, Liere P, Pianos A, Verwer RWH, Sluiter A, Huitinga I (2023). Disease stage-dependent changes in brain levels and neuroprotective effects of neuroactive steroids in Parkinson’s disease. Neurobiol Dis.

[31] Mathiesen SN, Lock JL, Schoderboeck L, Abraham WC, Hughes SM (2020). CNS transduction benefits of AAV-PHP.eB over AAV9 are dependent on Administration Route and Mouse strain. Mol Ther Methods Clin Dev.

[32] Messing A, Brenner M, Feany MB, Nedergaard M, Goldman JE (2012). Alexander disease. J Neurosci.

[33] Neumann H, Kotter MR, Franklin RJ (2009). Debris clearance by microglia: an essential link between degeneration and regeneration. Brain.

[34] Qi XR, Luchetti S, Verwer RWH, Sluiter AA, Mason MRJ, Zhou JN (2018). Alterations in the steroid biosynthetic pathways in the human prefrontal cortex in mood disorders: a post-mortem study. Brain Pathol.

[35] Qi XR, Verwer RWH, Bao AM, Balesar RA, Luchetti S, Zhou JN (2019). Human brain slice culture: a useful Tool to Study Brain disorders and potential therapeutic compounds. Neurosci Bull.

[36] Rademakers R, Baker M, Nicholson AM, Rutherford NJ, Finch N, Soto-Ortolaza A (2011). Mutations in the colony stimulating factor 1 receptor (CSF1R) gene cause hereditary diffuse leukoencephalopathy with spheroids. Nat Genet.

[37] Ravi VM, Joseph K, Wurm J, Behringer S, Garrelfs N, d’Errico P et al (2019) Human organotypic brain slice culture: a novel framework for environmental research in neuro-oncology. Life Sci Alliance 2. 10.26508/lsa.201900305 10.26508/lsa.201900305 PMC6599970 31249133

[38] Rosin JM, Marsters CM, Malik F, Far R, Adnani L, Schuurmans C (2021). Embryonic microglia interact with Hypothalamic Radial Glia during Development and Upregulate the TAM receptors MERTK and AXL following an insult. Cell Rep.

[39] Schwarz N, Hedrich UBS, Schwarz H, Dammeier PAH, Auffenberg N (2017). Human cerebrospinal fluid promotes long-term neuronal viability and network function in human neocortical organotypic brain slice cultures. Sci Rep.

[40] Sherman LS, Back SA (2008). A ‘GAG’ reflex prevents repair of the damaged CNS. Trends Neurosci.

[41] Skripuletz T, Hackstette D, Bauer K, Gudi V, Pul R, Voss E (2013). Astrocytes regulate myelin clearance through recruitment of microglia during cuprizone-induced demyelination. Brain.

[42] Sloane JA, Batt C, Ma Y, Harris ZM, Trapp B, Vartanian T (2010). Hyaluronan blocks oligodendrocyte progenitor maturation and remyelination through TLR2. Proc Natl Acad Sci U S A.

[43] Stoppini L, Buchs PA, Muller D (1991). A simple method for organotypic cultures of nervous tissue. J Neurosci Methods.

[44] Strawn JR, Ekhator NN, Geracioti TD (2001). In-use stability of monoamine metabolites in human cerebrospinal fluid. J Chromatogr B Biomed Sci Appl.

[45] Ting JT, Kalmbach B, Chong P, de Frates R, Keene CD, Gwinn RP (2018). A robust ex vivo experimental platform for molecular-genetic dissection of adult human neocortical cell types and circuits. Sci Rep.

[46] van der Knaap MS, Bugiani M (2017). Leukodystrophies: a proposed classification system based on pathological changes and pathogenetic mechanisms. Acta Neuropathol.

[47] Verwer RW, Hermens WT, Dijkhuizen P, ter Brake O, Baker RE, Salehi A (2002). Cells in human postmortem brain tissue slices remain alive for several weeks in culture. FASEB J.

[48] Verwer RW, Baker RE, Boiten EF, Dubelaar EJ, van Ginkel CJ, Sluiter AA (2003). Post-mortem brain tissue cultures from elderly control subjects and patients with a neurodegenerative disease. Exp Gerontol.

[49] Wisse LE, Penning R, Zaal EA, van Berkel CGM, Ter Braak TJ, Polder E (2017). Proteomic and metabolomic analyses of vanishing White Matter mouse astrocytes reveal deregulation of ER functions. Front Cell Neurosci.

[50] Wolf NI, Breur M, Plug B, Beerepoot S, Westerveld ASR, van Rappard DF (2020). Metachromatic leukodystrophy and transplantation: remyelination, no cross-correction. Ann Clin Transl Neurol.

[51] Yong Y, Hunter-Chang S, Stepanova E, Deppmann C (2021). Axonal spheroids in neurodegeneration. Mol Cell Neurosci.

[52] Zhang H, Jarjour AA, Boyd A, Williams A (2011). Central nervous system remyelination in culture–a tool for multiple sclerosis research. Exp Neurol.

[53] Zhang L, Verwer RWH, van Heerikhuize J, Lucassen PJ, Nathanielsz PW, Hol EM (2024). Progesterone receptor distribution in the human hypothalamus and its association with suicide. Acta Neuropathol Commun.

