# Supplementary material for: Human post-mortem organotypic brain slice cultures: a tool to study pathomechanisms and test therapies
Source: Acta Neuropathol Commun. 2024 May 31;12:83. doi: 10.1186/s40478-024-01784-1 (PMC11140981; doi:10.1186/s40478-024-01784-1)
Supplement: Supplementary file 1 — Additional file 1: Supplementary Method. Human post-mortem organotypic brain slice cultures. Detailed protocol for generating human post-mortem organotypic brain slice cultures. [file 40478_2024_1784_MOESM1_ESM.docx]

***Acta Neuropathologica Communications***

Human post-mortem organotypic brain slice cultures: a tool to study pathomechanisms and test therapies

Bonnie C. Plug^1,2^, Ilma M. Revers^1,2,†^, Marjolein Breur^1,2,†^, Gema Muñoz González^3^, Jaap A. Timmerman^4^, Niels R.C. Meijns^3^, Daniek Hamberg^1,2^, Jikke Wagendorp^1,2^, Erik Nutma^5^, Nicole I. Wolf^1,2^, Antonio Luchicchi^3^, Huibert D. Mansvelder^4^, Niek P. van Til^1,2^, Marjo S. van der Knaap^1,2,4^ & Marianna Bugiani^1,2,5^

^1^Department of Paediatrics and Child Neurology, Emma Children’s Hospital, Amsterdam University Medical Centres, Amsterdam Neuroscience, Cellular & Molecular Mechanisms, Meibergdreef 9, 1100 DD Amsterdam, The Netherlands

^2^Amsterdam Leukodystrophy Center, Emma Children’s Hospital, Amsterdam University Medical Centers, Amsterdam Neuroscience, Cellular & Molecular Mechanisms, Meibergdreef 9, 1100 DD Amsterdam, The Netherlands

^3^Department of Anatomy and Neurosciences, MS Center Amsterdam, Amsterdam University Medical Centres, VU University, Amsterdam Neuroscience, De Boelelaan 1108, 1081 HZ Amsterdam, The Netherlands

^4^Department of Integrative Neurophysiology, Center for Neurogenomics and Cognitive Research, VU University, Amsterdam Neuroscience, De Boelelaan 1085, 1081 HV Amsterdam, The Netherlands

^5^Department of Pathology, Amsterdam University Medical Centres, Amsterdam Neuroscience, Meibergdreef 9, 1100 DD Amsterdam, The Netherlands

**^†^These authors contributed equally to this work.**

**Corresponding author**: Marianna Bugiani, m.bugiani@amsterdamumc.nl

Supplementary Method

Human post-mortem organotypic brain slice cultures

Materials

Required items for slicing and culturing

1. Post-mortem human brain tissue specimen (see **Note 1**)
2. Thermo Fisher Microm HM 650V (store the vibratome tissue platform and cooling element at -20^o^C)
3. Stainless steel blades (Campden Instruments, 752-1-SS).
4. Super glue (Bison)
5. 4% Agar gel (see **Note 2**)
6. Styrofoam box with ice
7. Sterile 50 mL tubes
8. Sterile 50 mL syringe and BD® 20Gx3.5” spinal needle (for hCSF collection)
9. Disposable scalpels
10. Bent sanded spatula
11. Forceps
12. P200 Pipet and sterile pipet tips
13. Sterile petri dishes
14. 70% EtOH
15. Whatman filter paper
16. Phosphate-buffered saline (PBS)
17. 4% paraformaldehyde (PFA) in PBS (for slice fixation)
18. 0.05% sodium azide in PBS (for slice storage after fixation)
19. Sterile 6-well culture plates
20. Millicell cell culture plate membrane insert (30 mm, 0.4 µm pore size; Merck Millipore, PICM0RG50)
21. 0.22 µm filtration unit
22. Tissue culture laminar flow hood
23. Humidified cell culture incubator (37^o^C, 5% CO_2_)
24. Face mask (optional)

Dissection medium preparation

1. Hibernate-A (Invitrogen, A12475-01)
2. Penicillin-streptomycin (Invitrogen, 15140-122)

Normal slice culture medium preparation

1. Minimum essential medium (MEM, Thermo Fisher, 32360026)
2. Earle’s Balanced Salt Solution (EBSS, Thermo Fisher, 24010043)
3. Heat-inactivated horse serum (Thermo Fisher, 26050088)
4. 45% D-Glucose (Sigma-Aldrich, G8769)
5. GlutaMAX-I (100X) supplement (Thermo Fisher, 35050038)
6. Penicillin-streptomycin (Thermo Fisher, 15140-122)
7. Amphotericin B (Thermo Fisher, 15290018)

Experimental procedure

Part I. Prior to tissue collection

1. Prepare normal slice culture medium (see **Note 3**) by combining 50% MEM, 25% EBSS and 25% horse serum supplemented with 1% GlutaMAX-I, 1% penicillin-streptomycin, 1.25 μg/ml Amphotericin B and 2.6 mg/ml D-Glucose. Filter sterilize with a 0.22 μm filtration unit. The medium can be stored up to 4 weeks at 4^o^C.
2. Prepare dissection medium (see **Note 3**) by combining 100% Hibernate-A and 1% penicillin-streptomycin. The medium can be stored up to 4 weeks at 4^o^C.
3. Collect 25 ml dissection medium in a sterile tube (for tissue collection) and an empty sterile tube (for hCSF collection) and keep on ice.
4. Prepare 6-well culture plates (number of plates depends on the amount of slices needed and/or the amount of tissue that can be obtained) in a tissue culture laminar flow hood; add 1 ml slice culture medium per well and use sterile forceps to transfer the membrane inserts to the wells. Incubate at 37^o^C until further use.
5. Shortly before slicing, keep about 300 ml dissection medium at -20^o^C (but avoid complete freezing).
6. Clean the dissection workspace, all vibratome parts, blade and tools with 70% EtOH.
7. Assemble the vibratome and secure the blade in place. Keep the vibratome tissue platform and cooling element stored at -20^o^C until slicing.
8. Adjust the settings on the vibratome. For a Thermo Fisher Microm HM 650V, the following settings are suggested:
   1. Slice thickness = 300 µm
   2. Velocity = V8
   3. Frequency = 70 Hz
   4. Amplitude = 1
   5. Mode = single stroke

Part II. Tissue collection and processing

1. *Optional*: Wear a face mask during tissue collection until slice culturing to increase sterility.
2. Collect a tissue specimen (see **Note 4**) from the middle frontal gyrus (see **Note 5**) within 6h post-mortem (see **Note 6**) in about 25 ml ice-cold dissection (keep on ice throughout procedure).
3. If required, collect human cerebrospinal fluid (hCSF) using a sterile syringe and needle from the lateral ventricles in a sterile tube (keep on ice throughout procedure).
4. Transport within minutes to the dissection workspace.
5. Transfer the tissue specimen and dissection medium to a petri dish on ice. Carefully remove meninges and blood vessels using forceps.

*Optional*: Tissue processing can be performed in a flow hood for increased sterility.

1. Use sterile scalpels to cut out a tissue block of approximately 1.5x1.5cm containing about 40% cortex and 60% white matter. Keep the tissue block submerged in dissection medium on ice.

Part III. Tissue slicing

1. Collect the vibratome tissue platform and cooling element (stored at -20^o^C) and clean with 70% EtOH. Keep the tissue platform on ice and add the cooling element to the vibratome reservoir.
2. Use superglue to fix the 4% agar gel block on the tissue platform (see **Note 2**).
3. Use a sterile spatula and the blunt end of the scalpel (for support only) to collect the tissue block and remove excess medium using Whatman filter paper.
4. Glue the tissue block to the platform with the cortical surface facing the blade. Position the tissue platform in the vibratome reservoir.
5. Add ice-cold dissection medium to the reservoir to completely submerge the tissue (approx. 250 ml required).
6. Cut 300 µm-thick slices using the vibratome settings described above.

*Optional*: Following slice preparation, immediately fix several slices in 4% PFA to be used as a reference.

1. Transfer individual slices onto Millicell membrane culture inserts (see **Note 4**) in a culture plate using a spatula and the blunt end of the scalpel (for support only).

*Important*: make sure the slices do not touch the inner ring of the membrane insert.

1. Remove excess medium from the top of the inserts using a P200 pipet and sterile pipet tips. The inserts should be as dry as possible.
2. Incubate the slices at 37°C and 5% CO_2_.

*Important*: keep the culture plates in the incubator as much as possible throughout the slicing process.

1. In case of more tissue remaining and/or more slices desired, cut another tissue block of appropriate size and repeat tissue slicing steps 1 till 9.
2. If hCSF has been obtained, spin hCSF down for 5 min at 2200 rpm at RT and transfer the supernatant to a sterile tube. Depending on the volume of hCSF obtained, determine the amount of slices to be cultured with addition of hCSF. Prepare hCSF slice culture medium by adding appropriate volumes of hCSF to enriched slice culture medium in 1:1 dilution (v/v) (see **Note 7**). Replace the normal slice culture medium with 1 ml per well of the hCSF slice culture medium for the designated slices. Aliquot the remaining pure hCSF and store at -80^o^C until use.

Part IV. Slice culturing

1. Incubate the slices at 37^o^C and 5% CO_2_ up till 42 days *in vitro* (DIV) (see **Note 8**).
2. Change culture medium at 1 DIV and then three times a week at a regular interval; replace with 1 ml per well of either normal slice culture medium or hCSF slice culture medium.

*Important*: always freshly prepare hCSF slice culture medium at times of medium changes.

1. *Optional*: collect slice-conditioned culture medium at set time points in sterile 1.5 ml Eppendorf tubes. Store at -80^o^C until further use.
2. At endpoint, aspirate the culture medium (or collect if desired) and rinse membrane insert in 1 ml PBS. Fix slices in 2 ml 4% PFA in PBS (1 ml on top and 1 ml below membrane insert) for 1h at RT. Rinse 3x in 2 ml PBS. If required, store in 0.05% sodium azide in PBS until further use.

Notes

1. For the purpose of preparing human organotypic slice cultures, post-mortem human brain tissue can be obtained from donors with various clinical and genetic backgrounds (patients or controls) and of different ages, depending on the research question.
2. Prepare the 4% (w/v) agar gel by dissolving agar powder in boiling ultrapure water and pouring the solution in a petri dish to cool and form a gel. At the time of slicing, cut out an agar block of at least the same length as the tissue block and about 1 cm wide. Glue the agar block to the vibratome tissue platform to support the tissue specimen while slicing. This ensures the tissue is kept in place and hence improves the steadiness during slicing and consistency in thickness of slices.
3. Normal slice culture medium and dissection medium should be prepared in advance and ready at any given time.
4. Human post-mortem brain tissue specimens should be collected by dissecting perpendicular to the cortical surface. Tissue specimens should contain part of the cerebral cortex and subcortical white matter, with at least the same volume of white matter relative to cortex. Tissue specimens can be further dissected into tissue blocks of the desired size for processing with the vibratome. One membrane insert (Merck Millipore, PICM0RG50) fits one human organotypic slice with a maximum size of 1.5x1.5cm. This is roughly the average size of the slices cultured in the present study. If required, one membrane insert can fit multiple smaller sized-slices. The size of the dissected tissue specimen should be chosen depending on the required slice size and slice number.
5. Preparing and culturing slices from tissue specimens obtained from other brain regions than the middle frontal gyrus has not been tested in the current study. If abovementioned tissue specimen collection guidelines are followed, we expect similar outcomes when dissecting tissue specimens from other cortical areas of the brain.
6. Human post-mortem brain tissue collection should be performed within 6h post-mortem. Tissue quality and viability cannot be guaranteed at longer post-mortem delays.
7. hCSF was added to enriched slice culture medium in 1:1 dilution (v/v). Enriched slice culture medium consists of 100% normal slice culture medium supplemented with an additional 2.6 mg/ml of 45% D-Glucose, 1% GlutaMAX-I, 1% penicillin-streptomycin and 1.25 μg/ml Amphotericin B. hCSF was added to post-mortem human organotypic brain slice cultures derived from the same donor.
8. The developed human post-mortem organotypic slice culture method has been tested up till 42 days *ex vivo*. For this six week culture period, proof of viability has been supplied. This protocol might be applied for longer culture periods, in which case tissue quality and viability should be carefully characterized.
