# Supplementary figures and images for: Human post-mortem organotypic brain slice cultures: a tool to study pathomechanisms and test therapies
Source: Acta Neuropathol Commun. 2024 May 31;12:83. doi: 10.1186/s40478-024-01784-1 (PMC11140981; doi:10.1186/s40478-024-01784-1)

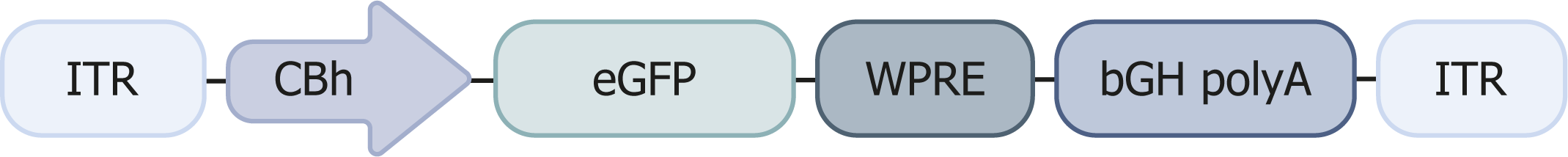

Supplement: Supplementary file 2 — Additional file 2: Supplementary Fig. 1. Schematic of the adeno-associated viral vector pCBh-eGFP. ITR = Inverted terminal repeat, CBh = CMV enhancer/chicken β-actin hybrid promoter, eGFP = enhanced green fluorescent protein, WPRE = Woodchuck Hepatitis Virus (WHV) posttranscriptional regulatory element, bGH polyA = bovine growth hormone polyadenylation signal. [file 40478_2024_1784_MOESM2_ESM.tif]

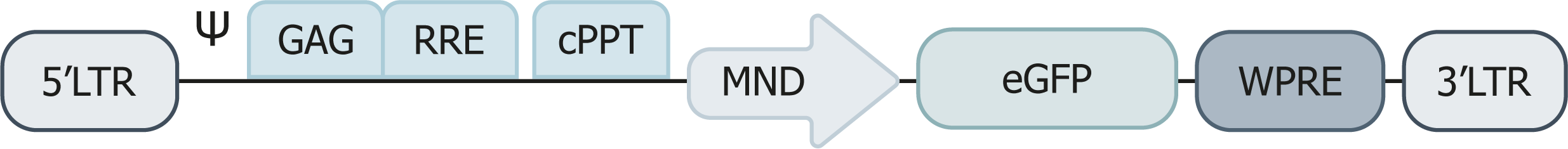

Supplement: Supplementary file 3 — Additional file 3: Supplementary Fig. 2. Schematic of the lentiviral vector pCCL_MND_EGFP_bPRE4_ SIN. LTR = Long-terminal repeat, psi sequence (Ψ) = RNA packaging signal, cPPT = central purine tract, GAG = HIV-1 GAG protein, RRE = Rev responsive element, MND = murine leukemia virus-derived promoter, eGFP = enhanced green fluorescent protein, WPRE = Woodchuck Hepatitis Virus (WHV) posttranscriptional regulatory element. [file 40478_2024_1784_MOESM3_ESM.tif]

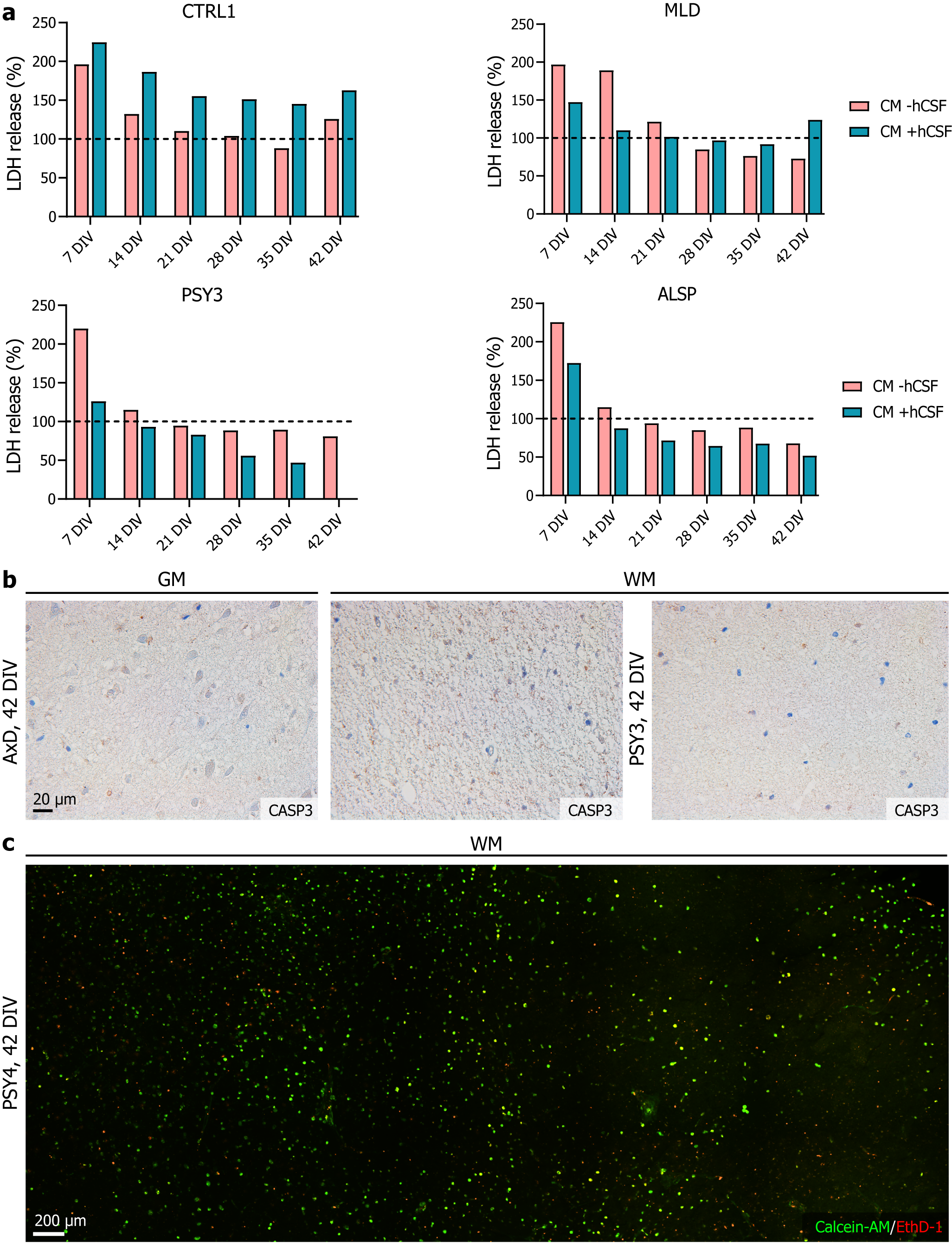

Supplement: Supplementary file 4 — Additional file 4: Supplementary Fig. 3. Long-term viability of HPMB-OSCs ex vivo. (a) LDH release in slice-CM of HPMB-OSCs obtained from donors CTRL1, PSY3, MLD, ALSP cultured without (pink) or with (blue) addition of hCSF. The dotted line represents the–hCSF and + hCSF medium controls, set at 100%. LDH release is presented as percentage of the corresponding medium control. Data are displayed as mean of technical replicates and hence no statistical analysis was performed. (b) Cleaved caspase-3 (CASP3) staining shows limited signs of apoptosis-related cell death at 42 DIV in patient and control slices in both the grey matter (GM) and white matter (WM). (c) LIVE/DEAD assay of HPMB-OSC obtained from PSY4 at 42 DIV with calcein-AM visualizing living cells and ethidium homodimer-1 (EthD-1) indicating dead cells. [file 40478_2024_1784_MOESM4_ESM.tif]

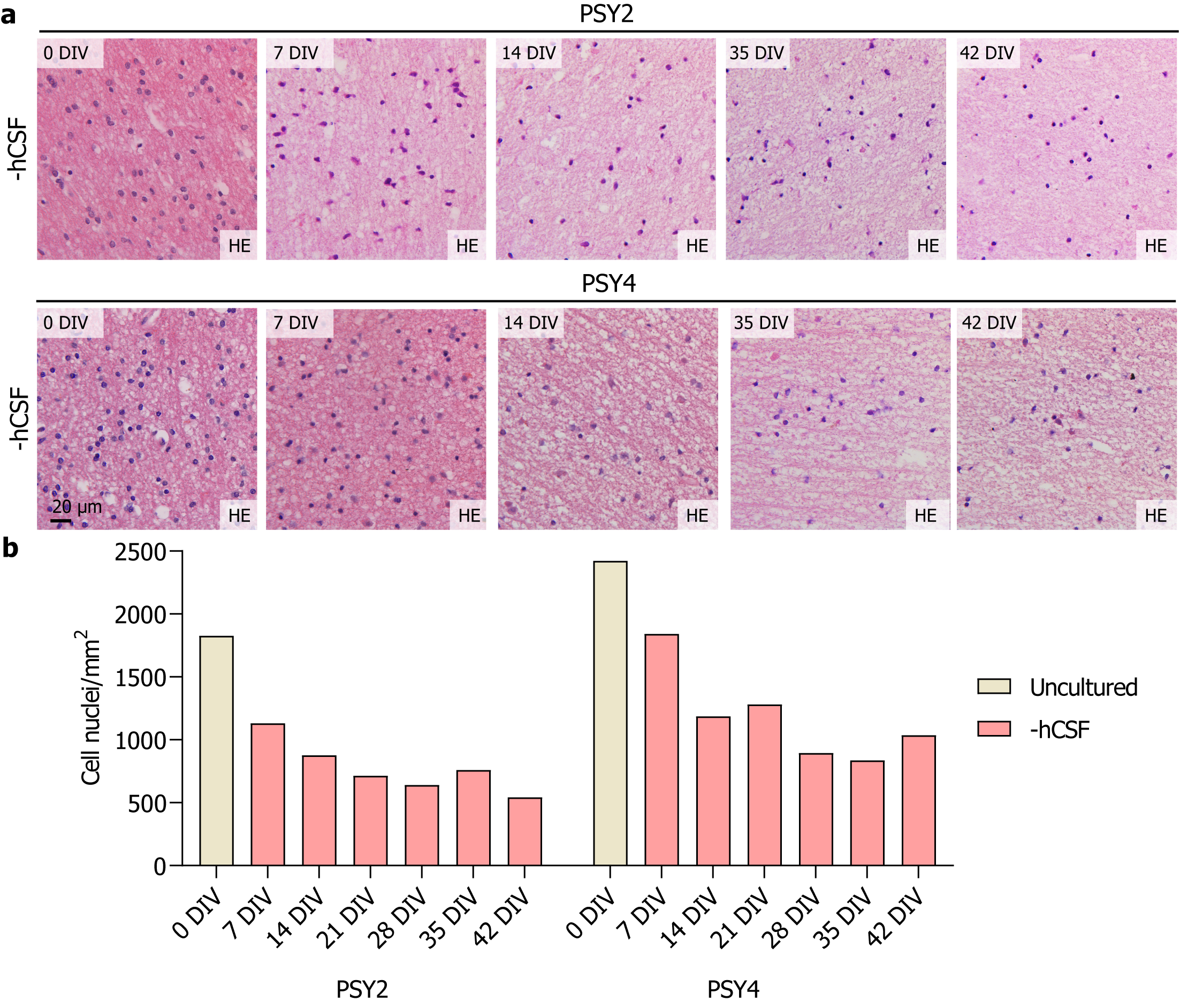

Supplement: Supplementary file 6 — Additional file 6: Supplementary Fig. 4. Cell density in control HPMB-OSC without hCSF supplementation throughout the culture period. (a) HE staining of HPMB-OSC of PSY2 (top) and PSY4 (bottom) cultured without hCSF supplementation at 0, 7, 14, 35, and 42 DIV shows a decrease in total cell number with increasing time in culture. Although the cell density is decreased after six weeks in culture, there is a considerable number of viable cells and the overall tissue structure remains well preserved. (b) Density of hematoxylin-stained cell nuclei in uncultured slices (0 DIV) and slices cultured up till 42 DIV without hCSF of PSY2 (left) and PSY4 (right). In both tissue cultures, the decrease in cell density is proportionally the largest in the first one to two weeks in culture, after which it relatively stabilizes. Data are displayed as mean of technical replicates and hence no statistical analysis was performed. [file 40478_2024_1784_MOESM6_ESM.tif]

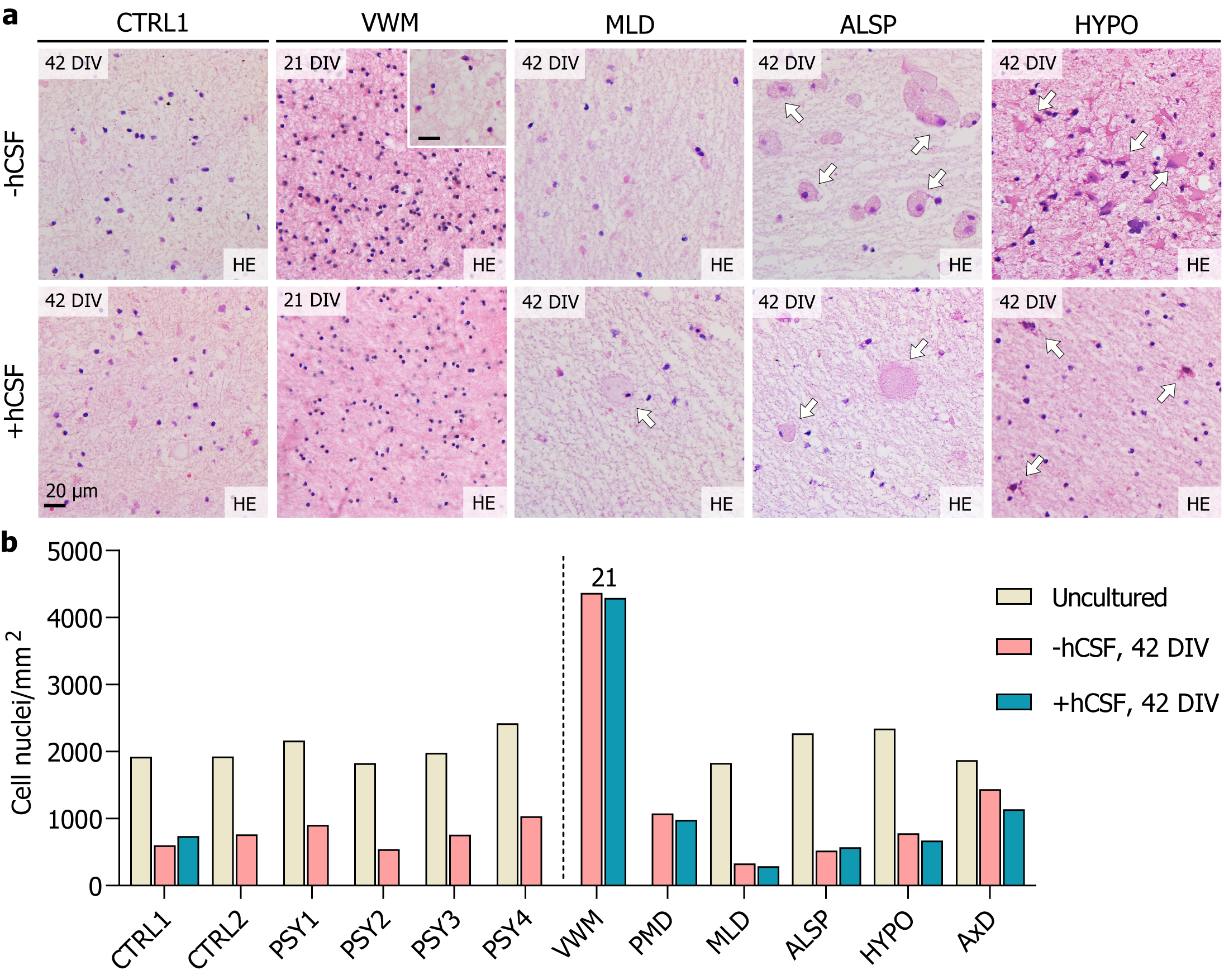

Supplement: Supplementary file 7 — Additional file 7: Supplementary Fig. 5. Cell density in control and patient-derived HPMB-OSC with and without hCSF supplementation. (a) HE staining of the white matter of control and patient-derived HPMB slices cultured without (top) and with (bottom) addition of hCSF at 42 DIV (and 21 DIV in the case of VWM) shows comparable total cell numbers. Disease-specific pathology is observed in slices cultured in both medium types, as indicated by white arrows. VWM patient slices show markedly increased cell density in the more preserved white matter close to the cortex (whole image) and paucity of cells and tissue rarefaction deeper in the white matter (insert) compared to control tissue. Both features are typically seen in VWM. MLD patient slices show enlarged, rounded microglia/macrophages, whereas ALSP patient slices show axonal spheroids and pigmented glia. Robust reactive astrogliosis is observed in HYPO patient slices cultured without hCSF, while addition of hCSF restricts the astrocytic response. (b) Density of hematoxylin-stained cell nuclei in control and patient-derived uncultured reference slices (beige) and slices cultured without (pink) and with (blue) hCSF for 42 DIV (and 21 DIV in the case of VWM). No reference slices were available for the VWM and PMD patient and no + hCSF data at 42 DIV was available for some donors due to small hCSF volumes obtained. The total cell density is decreased to similar levels in slices cultured with and without hCSF for 42 DIV. Data are displayed as mean of technical replicates and hence no statistical analysis was performed. [file 40478_2024_1784_MOESM7_ESM.tif]
