# Supplementary material for: Human post-mortem organotypic brain slice cultures: a tool to study pathomechanisms and test therapies
Source: Acta Neuropathol Commun. 2024 May 31;12:83. doi: 10.1186/s40478-024-01784-1 (PMC11140981; doi:10.1186/s40478-024-01784-1)
Supplement: Supplementary file 5 — Additional file 5: Supplementary Table 1. RNA and protein content of PFA-fixed HPMB-OSCs at 42 DIV. [file 40478_2024_1784_MOESM5_ESM.docx]

Supplementary Table

**Supplementary Table 1. RNA and protein content of PFA-fixed HPMB-OSCs at 42 DIV**

| **Donor ID** | **Time point** | **Sample weight (mg)** | **RNA content (ng/µL)** | **RNA purity (260/280 ratio)** | **Protein content (µg/µL)** |
| --- | --- | --- | --- | --- | --- |
| CTRL2 | 42 DIV | 130 | 177.68 | 1.78 | 0.95 |
| PSY1 | 42 DIV | 170 | 123.56 | 1.72 | 1.22 |
| MLD | 42 DIV | 100 | 260.33 | 1.88 | 0.63 |
| AxD | 42 DIV | 100 | 71.74 | 1.63 | 0.77 |

CTRL = control, DIV = days *in vitro*, PSY = psychiatric disorder, MLD = Metachromatic leukodystrophy, AxD = Alexander disease.
